# Beyond the Ångström Exponent: Probing Additional Information in Spectral Curvature and Variability of In Situ Aerosol Hyperspectral (0.3–0.7 μm) Optical Properties

**DOI:** 10.1029/2022JD037201

**Published:** 2022-11-03

**Authors:** Carolyn E. Jordan, Bruce E. Anderson, John D. Barrick, Dani Blum, Kathleen Brunke, Jiajue Chai, Gao Chen, Ewan C. Crosbie, Jack E. Dibb, Ann M. Dillner, Emily Gargulinski, Charles H. Hudgins, Emily Joyce, Jackson Kaspari, Robert F. Martin, Richard H. Moore, Rachel O’Brien, Claire E. Robinson, Gregory L. Schuster, Taylor J. Shingler, Michael A. Shook, Amber J. Soja, Kenneth L. Thornhill, Andrew T. Weakley, Elizabeth B. Wiggins, Edward L. Winstead, Luke D. Ziemba

**Affiliations:** ^1^ National Institute of Aerospace Hampton VA USA; ^2^ NASA Langley Research Center Hampton VA USA; ^3^ Science Systems and Applications Inc. Hampton VA USA; ^4^ Brown University Providence RI USA; ^5^ Christopher Newport University Hampton VA USA; ^6^ University of New Hampshire Durham NH USA; ^7^ University of California, Davis Davis CA USA; ^8^ William & Mary Williamsburg VA USA

**Keywords:** biomass burning aerosol, hyperspectral optical properties, in situ measurement techniques, FIREX‐AQ

## Abstract

Ångström exponents (*α*) allow reconstruction of aerosol optical spectra over a broad range of wavelengths from measurements at two or more wavelengths. Hyperspectral measurements of atmospheric aerosols provide opportunities to probe measured spectra for information inaccessible from only a few wavelengths. Four sets of hyperspectral in situ aerosol optical coefficients (aerosol‐phase total extinction, *σ*_ext_, and absorption, *σ*_abs_; liquid‐phase soluble absorption from methanol, *σ*_MeOH‐abs_, and water, *σ*_DI‐abs_, extracts) were measured from biomass burning aerosols (BBAs). Hyperspectral single scattering albedo (*ω*), calculated from *σ*_ext_ and *σ*_abs_, provide spectral resolution over a wide spectral range rare for this optical parameter. Observed spectral shifts between *σ*_abs_ and *σ*_MeOH‐abs_/*σ*_DI‐abs_ argue in favor of measuring *σ*_abs_ rather than reconstructing it from liquid extracts. Logarithmically transformed spectra exhibited curvature better fit by second‐order polynomials than linear *α*. Mapping second order fit coefficients (*a*_1_, *a*_2_) revealed samples from a given fire tended to cluster together, that is, aerosol spectra from a given fire were similar to each other and somewhat distinct from others. Separation in (*a*_1_, *a*_2_) space for spectra with the same *α* suggest additional information in second‐order parameterization absent from the linear fit. Spectral features found in the fit residuals indicate more information in the measured spectra than captured by the fits. Above‐detection *σ*_MeOH‐abs_ at 0.7 μm suggests assuming all absorption at long visible wavelengths is BC to partition absorption between BC and brown carbon (BrC) overestimates BC and underestimates BrC across the spectral range. Hyperspectral measurements may eventually discriminate BBA among fires in different ecosystems under variable conditions.

## Key Points

- Measured in situ atmospheric aerosol hyperspectral (<0.001 μm, resolution) optical properties in biomass burning plumes from 0.3 to 0.7 µm
- Log‐transformed spectra better fit by second‐order polynomials than Ångström exponents; measured spectra have details missed by fits
- Spectral curvature in absorption driven by myriad organic chromophores over full wavelength range

## 1 Introduction

Improved temporal and spatial resolution of remotely sensed observations of biomass burning are enabling models to better characterize fire behavior and forecast both fires and related air quality downwind. For example, high temporal resolution (5–15 min) Geostationary Operational Environmental Satellite (GOES) observations of fire radiative power (FRP) have led to a greater understanding of diurnal fire behavior (Schmidt, 2019; Wiggins et al., 2020), while finer spatial resolution better captures horizontal fire spread (e.g., Schroeder et al., 2014, 2016). Typically, improvements in temporal resolution are made at the expense of spatial resolution and vice versa, for example, 5–15 min GOES FRP observations are made at a spatial scale of 2 km (Wiggins et al., 2020), whereas low earth polar orbiting sensors in a sun‐synchronous orbit such as Visible Infrared Imaging Radiometer Suite (VIIRS) provide a spatial scale of 375 m approximately twice per day (e.g., Schroeder et al., 2014). However, science working groups engaged in planning future satellite missions evaluate the trade space not just in terms of temporal and spatial resolution, but in terms of spectral range and resolution as well (e.g., Fishman et al., 2012).

Space‐based sensors measuring aerosol properties such as Moderate‐Resolution Imaging Spectrometer, Medium Resolution Imaging Spectrometer, and VIIRS have relied on some number of wavelength bands (≤15, FWHM ∼20 to 50 nm) spanning the visible to infrared wavelength range (see specifics on channels for each sensor available from https://ladsweb.modaps.eosdis.nasa.gov/missions-and-measurements/). However, sensors on track for launch over the next 2 years will have hyperspectral measurement capabilities. Tropospheric Emissions: Monitoring of Pollution in geostationary orbit will have hyperspectral resolution (0.57 nm) over two wavelength ranges: 290–490 and 540–740 nm (Zoogman et al., 2017). Phytoplankton, Aerosol, Cloud, ocean Ecosystem in a sun‐synchronous orbit will have hyperspectral resolution (5 nm) over 340–890 nm from its Ocean Color Imager and 2–4 nm resolution over 385–770 nm from its SPEXone polarimeter (Werdell et al., 2019). This evolution in technology from individual band sets to hyperspectral measurements of atmospheric aerosol properties by space‐based sensors motivates the development of hyperspectral measurement techniques for in situ atmospheric measurements of them as well. In situ measurements can be directly related to simultaneous measurements of aerosol physicochemical characteristics to improve interpretations of remote sensing retrievals and provide a more complete understanding of the role of varying ambient aerosol populations on visibility, photolysis rates, and radiative forcing.

Historically, the spectral behavior of aerosol extinction, scattering, and absorption (each can be represented by *p*(*λ*) as in Equation 1) have been reconstructed from some number (≥2) of discrete measured wavelengths, assuming the spectra can be described by a power law (e.g., Ångström, 1929; Eck et al., 1999; Moosmüller & Chakrabarty, 2011),

(1) $$p\left(\lambda \right)=\beta \lambda^{-\alpha }$$

where wavelength (*λ*) is in units of μm, *β* is the value of *p* at 1 μm, and *α* is the Ångström exponent. *α* provides a single parameter to describe the power law wavelength dependence in Equation 1, that is, the negative slope of a wavelength‐independent line in natural logarithm space,

(2) $$\alpha =-\frac{d\mathrm{LN}\left(p\left(\lambda \right)\right)}{d\mathrm{LN}\left(\lambda \right)}$$

This is an especially convenient parameterization as the assumption of wavelength‐independence of *α* allows for the calculation of the spectral range of interest from measured values at any two (or more) wavelengths.

However, observations in the ambient atmosphere have revealed curvature in the logarithmically transformed extinction (or aerosol optical depth) spectra (e.g., Eck, Holben, Dubovik, et al., 2001; Eck, Holben, Ward, et al., 2001; Eck et al., 1999, 2003a, 2003b; Kaskaoutis et al., 2010, 2011; Kaufman, 1993; King & Byrne, 1976; King et al., 1978; O’Neill et al., 2001; Rao & Niranjan, 2012; Reid et al., 1999; Schuster et al., 2006) that has been described in terms of a second‐order polynomial (e.g., Eck et al., 1999, 2001b; Schuster et al., 2006).

(3) $$\mathrm{LN}\left(p\left(\lambda \right)\right)=a_{0}+a_{1}\mathrm{LN}\left(\lambda \right)+a_{2}{\left(\mathrm{LN}\left(\lambda \right)\right)}^{2}$$

More recently Jordan, Stauffer, Lamb, Hudgins, et al. (2021) and Jordan, Stauffer, Lamb, Novak, et al. (2021) have reported second‐order polynomials provide a better fit to ambient in situ aerosol hyperspectral observations of absorption as well as extinction using a similar set of measurements to those in this work. The derivative of Equation 3 can be used to relate the linear and second‐order coefficients (*a*_1_ and *a*_2_, respectively) to *α* at the characteristic wavelength of the measurement range, *λ*_ch_ (see Jordan, Stauffer, Lamb, Hudgins, et al., 2021; Jordan, Stauffer, Lamb, Novak, et al., 2021 for more details),

(4) $$\frac{d\mathrm{LN}\left(p\left(\lambda \right)\right)}{d\mathrm{LN}\left(\lambda \right)}=-\alpha =a_{1}+2a_{2}\mathrm{LN}\left(\lambda_{ch}\right)$$

The concept of *λ*_ch_ is important because it explicitly accounts for curvature, that is, the values of *α*, *a*_1_, and *a*_2_ all depend on the wavelengths used to calculate them. This wavelength dependence requires the use of proper wavelength units (μm) as specified in Equation 1, that is, nm cannot be interchanged for μm in this set of equations. Equation 4 shows that *α* maps into a line in the two‐dimensional space defined by *a*_1_ and *a*_2_.

It is important to note that both parameterizations (Equations 2 and 3) are empirical. They are not derived from first principles of underlying physical or chemical properties of ambient aerosols. Hence, other mathematical approaches may yet be found that provide a better description of the relationships between aerosol optical, microphysical, and chemical properties. Analogous to improvements in measured temporal and spatial resolution, one may expect that improvements in measured spectral resolution will lead to advances in understanding these relationships. This work explores that potential using biomass burning data collected aboard the ground‐based NASA Langley Research Center's (LaRC) Mobile Aerosol Characterization (MACH‐2) laboratory (Figure S1 in Supporting Information S1) during the Fire Influence on Regional to Global Environments and Air Quality (FIREX‐AQ) campaign.

As reflected by the title “Beyond the Ångström Exponent,” this study is intended to motivate consideration of approaches that can be applied to extract more information from spectral variability when hyperspectral data are available. The discussion in Section 4 provides a synthesis of recent work related to biomass burning organic aerosols (BBOAs) to explain why hyperspectral information may be required, along with new mathematical approaches, to more fully characterize BBOA chromophores (light absorbing molecular structures) and their evolution in the ambient environment. More needs to be done to develop in situ hyperspectral measurement techniques for the ambient atmosphere so that such measurements become routine. We hope this work will help to inspire that development.

## 2 Methods

### 2.1 MACH‐2 Deployment for FIREX‐AQ

FIREX‐AQ was a two‐part study conducted from July to September 2019 with deployments of multiple platforms across the United States to study wildfires in the west and agricultural fires in the southeast. MACH‐2 (Figure S1 in Supporting Information S1) deployed only for the western portion of the campaign. It is a customizable ground sampling laboratory that was equipped by Langley researchers to primarily measure aerosol optical and microphysical measurements, with important ancillary gas phase measurements including carbon monoxide (CO), carbon dioxide (CO_2_), water vapor (H_2_O), ozone (O_3_), nitrogen dioxide (NO_2_), nitrous acid (HONO), and nitric acid (HNO_3_). MACH‐2 hosted research teams from Brown University, Christopher Newport University, and the University of New Hampshire. A study of HONO observations at surface sites downwind from several fires has been published by the MACH‐2 team (Chai et al., 2021). This work focuses on the in situ aerosol hyperspectral (0.3–0.7 μm) extinction and absorption measurements from five of the eight fires sampled across six states.

**Figure 1:**
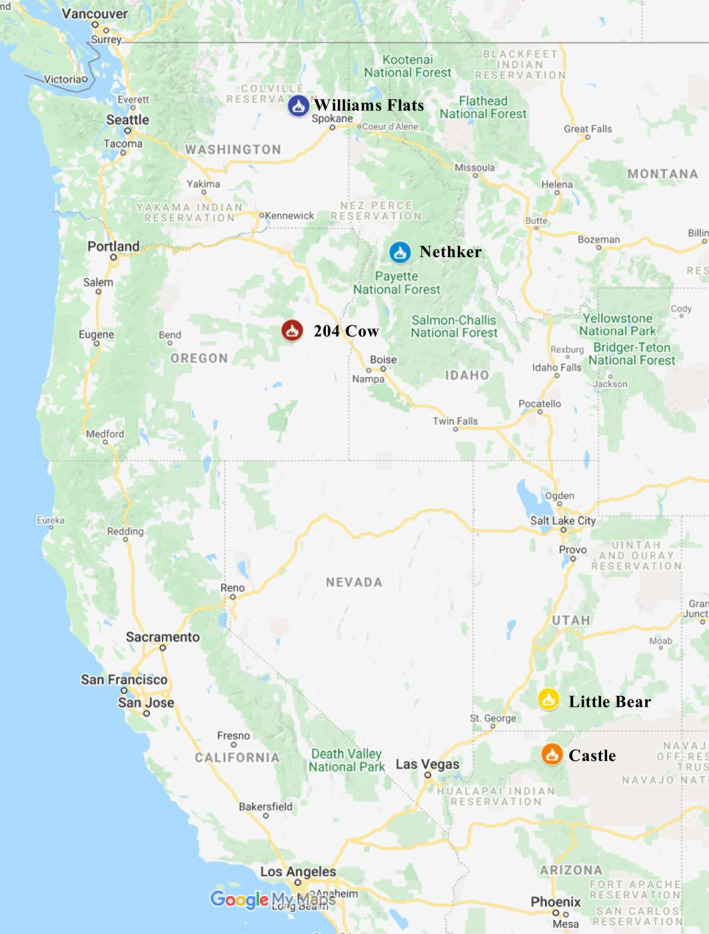
Map of fires sampled for this study. The fire symbols are color coded to match color scheme of data shown in Figures 2, 3 and 7. Map created using My Maps in Google Maps.

Data from five fires are examined here: Williams Flats (#3) near Spokane in eastern Washington, Nethker (#4) in central Idaho, Little Bear (#5) near Bryce Canyon in southern Utah, Castle (#6) near Jacobs Lake in northern Arizona, and 204 Cow (#7) near Prairie City in eastern Oregon (Figure 1). The numbers in parentheses indicate the order in which these fires were sampled over the course of the campaign (Table S1 in Supporting Information S1) and are used in some figures in place of fire names. The other three fires not presented either had limited data or were excluded due to generator exhaust contamination of the filter samples (see Section S2 in Supporting Information S1 for details).

For any given smoke plume there are four fundamental drivers causing the variability in the observations: (a) the fuels consumed, (b) the combustion phase (smoldering‐to‐flaming), (c) fire intensity and surface geometry, and (d) atmospheric processing as smoke travels away from the source. Fire intensity and the geometry of the surface burning (ranging from a large broad patch nearly as wide as it is long to a narrow fire front line along a burned perimeter) are critically important factors for vertical lofting and plume injection height, plume density (thick or thin) and geometry (spatial dimensions), and subsequent atmospheric processing that can differ from plume edges to plume core on the basis of light characteristics and locally variable oxidants across the plume spatial dimensions (e.g., Garofalo et al., 2019; Hodshire et al., 2019; Peterson et al., 2022; Wang et al., 2021). Additionally, meteorological conditions (e.g., Peterson et al., 2022) and the chemical composition of the background air (e.g., Hodshire et al., 2019) are important factors in atmospheric processing of the plume. The complexity of plume dynamics and evolution that arise from intense fires and surface burning geometry are beyond the scope of this work because MACH‐2 is not the appropriate platform to investigate these dynamics. Smoke that remains at the surface is either produced by low temperature combustion processes (as will be discussed further in Section 4) or is trapped in a shallow boundary layer such as can occur under a nocturnal inversion. In this work, we will use a simplified framework to discuss the differences observed in our data arising from (a) fuels consumed, (b) the fire state (by which we mean the combustion phase and completeness of combustion), and (c) atmospheric processing (here, simplified to the distance of MACH‐2 from the fire front).

Various metrics are used to quantify these drivers, and Table 1 provides a few examples. The approximate distance of MACH‐2 from the fire front, the daily sum of hourly FRP (in MW) observed from GOES, and fuels predominantly burned were estimated by the FIREX‐AQ Fuel2Fire team (Table 1). The modified combustion efficiency (MCE = ΔCO_2_/(ΔCO + ΔCO_2_)) calculated from the excess filter mean volume mixing ratios (Δ*X* = *X*_plume_ − *X*_background_) is often used to distinguish relative contributions from flaming and smoldering combustion (e.g., Hobbs et al., 2003). However, the diurnal variability of CO_2_ due to its uptake and respiration by plants made it challenging to accurately quantify background values of CO_2_ from the MACH‐2 data set given the timescale of sampling, that is, parked within a plume typically for many hours at a time. Instead, ΔBC/ΔCO (Table 1 and Table S3 in Supporting Information S1) is used an alternate metric for those conditions (e.g., Selimovic et al., 2019, and references therein). BC is produced only by flaming (Selimovic et al., 2019), while CO is indicative of incomplete combustion (i.e., smoldering). Black carbon (BC) was measured using a Multi‐Angle Aerosol Absorption Photometer (Thermo Fisher Scientific Inc., Franklin, MA; Table S3 in Supporting Information S1). CO was measured using a Los Gatos Research CO/CO_2_ analyzer (Los Gatos Research, San Jose, CA). Finally, ΔBC/ΔPM_2.5_ (Table 1 and Table S3 in Supporting Information S1) is also explored as another metric of this kind where PM_2.5_ was measured with a model T640 PM mass monitor (Teledyne API, San Diego, CA). Differences between the fires represented by these metrics will be discussed in Section 3.1.

**Table 1:** Filter Number, Fire Number and Name, Approximate Distance of MACH‐2 From Fire Front (km), Daily Sum of GOES Fire Radiative Power (MW), ΔBC/ΔCO, ΔBC/ΔPM_2.5_, and Predominant Fuels Consumed by Fire

Filter #	Fire #:name	∼Dist. from fire front (km)	Daily sum GOES FRP (MW)	ΔBC/ΔCO (μg m^−3^/ppmv)	ΔBC/ΔPM_2.5_ (μg m^−3^/μg m^−3^)	Fuels^a^
1	3:Wms Flts	26.7	6.55E+05	10.53	0.041	Grasses, Douglas Fir, Ponderosa Pine, Shrublands
2	3:Wms Flts	18.6	3.82E+05	13.01	0.050	Douglas Fir, Ponderosa Pine, Grasslands
3	3:Wms Flts	21.6	3.82E+05	10.59	0.040	Douglas Fir, Ponderosa Pine, Grasslands
4	3:Wms Flts	21.6	3.82E+05	11.17	0.042	Douglas Fir, Ponderosa Pine, Grasslands
5	3:Wms Flts	21.6	6.94E+05	2.51	0.017	Douglas Fir, Ponderosa Pine, Grasslands
6	3:Wms Flts	21.7	7.36E+05		0.040	Douglas Fir, Ponderosa Pine, Grasslands
7	3:Wms Flts	9.3	7.36E+05	12.63	0.051	Douglas Fir, Ponderosa Pine, Grasslands
8	3:Wms Flts	13.4	2.37E+06	9.88	0.055	Douglas Fir, Ponderosa Pine, Grasslands
9	4:Nethker	2.2		0.32	0.007	Douglas Fir, Ponderosa Pine, Subalpine Fir
10	4:Nethker	2.2		0.39	0.009	Douglas Fir, Ponderosa Pine, Subalpine Fir
11	4:Nethker	2.2		0.49	0.010	Douglas Fir, Ponderosa Pine, Subalpine Fir
12	4:Nethker	1.7		0.54	0.012	Douglas Fir, Ponderosa Pine, Subalpine Fir
13	4:Nethker	1.8		0.72	0.013	Douglas Fir, Ponderosa Pine, Subalpine Fir
14	4:Nethker	1.8		0.57	0.007	Douglas Fir, Ponderosa Pine, Subalpine Fir
15	4:Nethker	1.8		0.33	0.006	Douglas Fir, Ponderosa Pine, Subalpine Fir
16	4:Nethker	1.8		0.27	0.006	Douglas Fir, Ponderosa Pine, Subalpine Fir
17	4:Nethker	2.5		0.62	0.011	Douglas Fir, Ponderosa Pine, Subalpine Fir
18	4:Nethker	2.5		0.56	0.009	Douglas Fir, Ponderosa Pine, Subalpine Fir
19	4:Nethker	2.5		0.56	0.009	Douglas Fir, Ponderosa Pine, Subalpine Fir
20	4:Nethker	2.1		0.11	0.004	Douglas Fir, Ponderosa Pine, Subalpine Fir
21	4:Nethker	2.0		0.20	0.007	Douglas Fir, Ponderosa Pine, Subalpine Fir
22	4:Nethker	2.0		0.22	0.006	Douglas Fir, Ponderosa Pine, Subalpine Fir
23	4:Nethker	2.0		0.27	0.007	Douglas Fir, Ponderosa Pine, Subalpine Fir
24	5:Ltl. Bear	3.4	4.59E+04	11.58	0.061	Fir‐Gambel Oak, Ponderosa Pine Savanna
25	5:Ltl. Bear	3.6	3.25E+03	1.58	0.025	Fir‐Gambel Oak, Ponderosa Pine Savanna
26	5:Ltl. Bear	3.6	3.25E+03	1.45	0.019	Fir‐Gambel Oak, Ponderosa Pine Savanna
27	6:Castle	10.1	1.83E+03	5.32	0.036	Ponderosa Pine, Douglas Fir, Oak, Shrubland
28	6:Castle	10.1	1.83E+03	3.29	0.030	Ponderosa Pine, Douglas Fir, Oak, Shrubland
29	6:Castle	10.1	1.83E+03	3.12	0.029	Ponderosa Pine, Douglas Fir, Oak, Shrubland
30	6:Castle	11.1	1.83E+03	5.01	0.032	Ponderosa Pine, Douglas Fir, Oak, Shrubland
31	7:204 Cow	6.5	1.13E+04	3.30	0.032	Douglas Fir, Engelmann Spruce, Ponderosa Pine
32	7:204 Cow	6.9	1.13E+04	2.64	0.028	Douglas Fir, Engelmann Spruce, Ponderosa Pine
33	7:204 Cow	6.9	1.13E+04	2.41	0.030	Douglas Fir, Engelmann Spruce, Ponderosa Pine
34	7:204 Cow	3.5	3.88E+04		0.020	Douglas Fir, Engelmann Spruce
35	7:204 Cow	3.5	3.88E+04		0.015	Douglas Fir, Engelmann Spruce
36	7:204 Cow	3.5	3.88E+04		0.015	Douglas Fir, Engelmann Spruce
37	7:204 Cow	4.3	3.88E+04		0.017	Douglas Fir, Engelmann Spruce
38	7:204 Cow	4.3	3.88E+04		0.017	Douglas Fir, Engelmann Spruce
39	7:204 Cow	4.3	3.88E+04		0.021	Douglas Fir, Engelmann Spruce
40	7:204 Cow	4.3	3.88E+04		0.020	Douglas Fir, Engelmann Spruce

*Note.* Gray font used to highlight the only filter sample collected in predominantly background conditions (FN‐5, see Table S3 in Supporting Information S1).

^a^ Based on the Fuel Characteristic Classification System 2014 30 m fuels database.

### 2.2 SpEx Extinction Coefficient Spectra, *σ*_ext_(*λ*), Measurements

The custom Spectral Aerosol Extinction (SpEx) instrument was designed to measure extinction coefficient spectra, *σ*_ext_(*λ*), of in situ ambient atmospheric aerosols and it has been described previously by Jordan et al. (2015) and Jordan, Stauffer, Lamb, Hudgins, et al. (2021). In short, it is comprised of a White‐type optical cell with a 17 L internal volume and 39.4 m optical path connected via fiber optics to a UV5000 system (Cerex Monitoring Solutions, LLC, Atlanta, GA) containing a 150 W xenon lamp light source (Cerex P/N CRX‐X150W) integrated with an Ocean Optics, Inc. (Dunedin, FL) QE65Pro 16‐bit spectrometer. Hyperspectral (∼0.0008 μm resolution) *σ*_ext_(0.3–0.7 μm) are acquired every 4 min with a measurement error of ∼± 5 Mm^−1^ over the full spectral range.

Here, *σ*_ext_ spectra collected over each filter sampling time were averaged for comparison with the filter absorption spectra data sets (see Table S1 in Supporting Information S1 for averaging intervals). Occasionally, the light intensity saturated the spectrometer (usually around 0.47 μm, but for the first Williams Flats filter an alignment issue led to saturation in three bands of wavelengths ≥0.61 μm). The affected wavelengths were removed from the mean *σ*_ext_ spectra (see Jordan, Stauffer, Lamb, Hudgins, et al., 2021 for further discussion of this issue). These small data gaps did not affect the curve fits that will be discussed in Section 3.3.

### 2.3 Teflon Filter FT‐IR Analyses and Subsequent Soluble Absorption Spectra Measurements

Three sets of 47 mm filters were collected in parallel behind a PM_2.5_ cyclone, sampling from the same inlet and sampling manifold as the rest of the instrument complement in MACH‐2. One set of Teflon filters (Teflo, 2 mm, Pall Corp.) were pre‐weighed, labeled, and stored individually prior to sampling. The other two sets were pre‐baked quartz filters (Pallflex Tissuquartz Air Monitoring Filters, Pall Corp.) also stored individually in petri dishes before and after sampling (described in Section 2.4).

The Teflon filter set was analyzed by FT‐IR at the University of California, Davis, CA, using a Tensor II (Bruker Optics, Billerica, MA), infrared spectrometer equipped with a liquid nitrogen‐cooled mercury cadmium telluride detector (see Section S4 in Supporting Information S1 for details). Spectra are baseline corrected prior to peak‐fitting to estimate concentrations of organic functional groups that include aliphatic C‐H (aCH), alcohol OH (aCOH), carboxylic acids (COOH), and non‐acid carbonyls (naCO) (Reggente et al., 2019 and references therein).

After FT‐IR analysis, the filters were shipped to LaRC for extraction and analysis in a liquid waveguide capillary cell (LWCC) to obtain both water‐soluble (Milli‐Q 18MW deionized (DI) water) and methanol‐soluble (MeOH, spectrophotometric‐grade, ≥99.9%) absorption coefficient spectra (*σ*_DI‐abs_(*λ*) and *σ*_MeOH‐abs_(*λ*), respectively) of soluble aerosol components. The methodology for these analyses is described in detail in Jordan, Stauffer, Lamb, Novak, et al. (2021) with some minor adjustments made here (Section S4 in Supporting Information S1). As explained in Section S4 in Supporting Information S1, there are no *σ*_DI‐abs_ spectra for the Williams Flats fire due to laboratory handling problems. All spectra were blank corrected and only the portion of the spectrum above the LLOD was retained for analysis. The *σ*_MeOH‐abs_ spectra were above detection across the entire 0.3–0.7 μm wavelength range, with absorbance typically 2–3 orders of magnitude greater than the LLOD at 0.7 μm. In contrast, the *σ*_DI‐abs_ spectra were not always above detection at longer visible wavelengths. Error propagation of uncertainty in these measurements resulted in an uncertainty of about ±30%.

Note, the approach to analyzing the soluble absorption in this study differs from that typically used to estimate total brown carbon (BrC) absorption coefficients for ambient aerosols. In prior studies, a filter is extracted sequentially first with DI then MeOH. This allows for the DI extract to be analyzed for DI‐soluble ions using ion chromatography, as well as for absorption by chromophores present in bulk solution with the sum of *σ*_DI‐abs_(*λ*) and *σ*_MeOH‐abs_(*λ*) representing the total absorption of chromophores extracted into solution (e.g., Hecobian et al., 2010; X. Zhang et al., 2013; Y. Zhang et al., 2017; Zeng et al., 2022). The total absorption of the bulk solution chromophores is then corrected to account for enhanced absorption of ambient particles in the Mie regime with a typical correction of ∼2 applied to BrC(0.365 μm) (e.g., Liu et al., 2013; Washenfelder et al., 2015; Y. Zhang et al., 2017). Studies that evaluate BrC contributions to total absorption across a broader spectral range may take an approach of applying the factor of 2 correction at 0.365 μm, then use absorption Ångström exponents (*α*_abs_) to calculate BrC contributions at other wavelengths (e.g., Y. Zhang et al., 2017). More recent work has shown that the correction factor determined from Mie theory varies as a function of wavelength over the 0.3–0.7 μm range (Zeng et al., 2022). Either way, this conversion relies on several assumptions regarding particle size distributions, refractive indices, mixing state, etc., such that this type of correction may be expected to alter the wavelength dependence from the bulk solution spectrum to the approximated ambient aerosol spectrum. In this work, it is the measured spectral characteristics that are of interest. Hence, the measured bulk solution spectra are presented here without transformation to approximate equivalent ambient aerosol spectra of the extracted chromophores.

### 2.4 Pre‐Baked Quartz Filter Total Absorption Coefficient Spectra, *σ*_abs_(*λ*), and Measurements

We built a prototype filter holder (Figure S4 in Section S5 in Supporting Information S1) coupled via fiber optics to the SpEx light source to measure hyperspectral total absorption spectra similar to the methodology used for Particle Soot Absorption Photometer (Radiance Research, Seattle, WA) and Tricolor Absorption Photometer (Brechtel, Hayward, CA) instruments (e.g., Bond et al., 1999; Ogren et al., 2017). A separate spectrometer was purchased to enable online measurements independent of the SpEx spectrometer during the campaign, but the new spectrometer failed in the field. Nonetheless, it was possible to couple the prototype to both the SpEx lamp and spectrometer post‐mission in the laboratory at LaRC to measure *σ*_abs_(*λ*) from 1 cm punches from one set of the quartz filters (see Sections S4 and S5 in Supporting Information S1). Error propagation was used to estimate the uncertainty in the mean spectrum for each filter. The intensity of light below 0.35 μm was limited in the prototype configuration (Section S5 in Supporting Information S1). This will be corrected for future studies. Here, the *σ*_abs_ spectra were truncated to a range of 0.35–0.7 μm.

## 3 Results

### 3.1 Differences in Fire Observations

#### 3.1.1 Fuels

The three northernmost fires (Williams Flats, Nethker, and 204 Cow, Figure 1) exhibited some overlap in the fuels burned; for example, Douglas Fir was the dominant fuel for all three (Table 1), while Ponderosa Pine was also prevalent at both Williams Flats and Nethker. The ecosystems of the two southern fires (Little Bear and Castle, Figure 1) also had Ponderosa Pine present but differed in some of the other dominant species (e.g., oak, savanna, and shrubland species). Grasslands were important at Williams Flats, Engelmann Spruce at 204 Cow. Subsequent references to northern versus southern fires in this work are intended as a shorthand to distinguish between the five fires observed on the basis of ecosystem fuels.

#### 3.1.2 Distance Downwind

The distance downwind from the fire where samples could be collected was constrained by the proximity of roads to the fire. At Williams Flats MACH‐2 ranged from 9.3 to 26.7 km from the fire front, while at Nethker it was within 2.5 km for all samples (Table 1). At Little Bear MACH‐2 was ∼3.5 km distant, while it ranged from 3.5 to 6.9 km downwind at 204 Cow, and 10.1–11.1 km at Castle (Table 1). However, it must be stressed that these distance estimates cannot account for localized pockets of fire that were nearby, often very close to the road during sampling at Nethker and 204 Cow. Nor do these distances capture local firefighting activities creating fire breaks where those activities may have led to flaming/smoldering conditions that differed from the main fire front. In particular, such fire‐fighting activities were close by on the first day of sampling at 204 Cow, but not the second.

#### 3.1.3 Fire Intensity

Characterizing fire intensity proved to be challenging. First, MCE could not be accurately determined due to the MACH‐2 sampling strategy (see Section 2.1). Second, FRP could not be ascertained for all five fires because the Nethker fire was not observed by GOES (Table 1). This was due both to extensive cloud cover on 11 August (UTC, see Table S1 in Supporting Information S1) and to the low temperature fire being below detection for the GOES sensor even when cloud cover was limited for the remaining days MACH‐2 sampled this fire. This indicated that Nethker provided the smoldering end of the observed range in fire intensity with Williams Flats and its generation of towering pyrocumulonimbus clouds (Peterson et al., 2022) the most intense flaming end of the range, with the other fires in between. The ΔBC/ΔCO metric used to characterize the degree of flaming proposed by Selimovic et al. (2019) supports this inference. The highest values were found in the Williams Flats smoke and the smallest in Nethker smoke (Table 1).

Normalizing the excess (i.e., smoke) mass concentration of BC by the excess mixing ratio of CO accounts for the effects of dilution of the plume when comparing samples either from the same fire or across fires. Selimovic et al. (2019) describe ΔBC/ΔCO as an inert tracer, since atmospheric processing on the scale of a plume is too slow to affect the concentrations of BC and CO beyond dilution after emission. In contrast, they refer to ΔPM_2.5_/ΔCO as a dynamic tracer because PM_2.5_ is subject to various atmospheric processes such as evaporation, condensation, coagulation, and photooxidative processes leading to changes with respect to CO post‐emission. Unlike airborne sampling that allows for Lagrangian sampling, the road limitation of MACH‐2 did not provide a sample set that could be used to probe the evolution of aerosol for a plume from a given fire (i.e., samples from a given plume were not obtained at a series of locations downwind).

**Figure 2:**
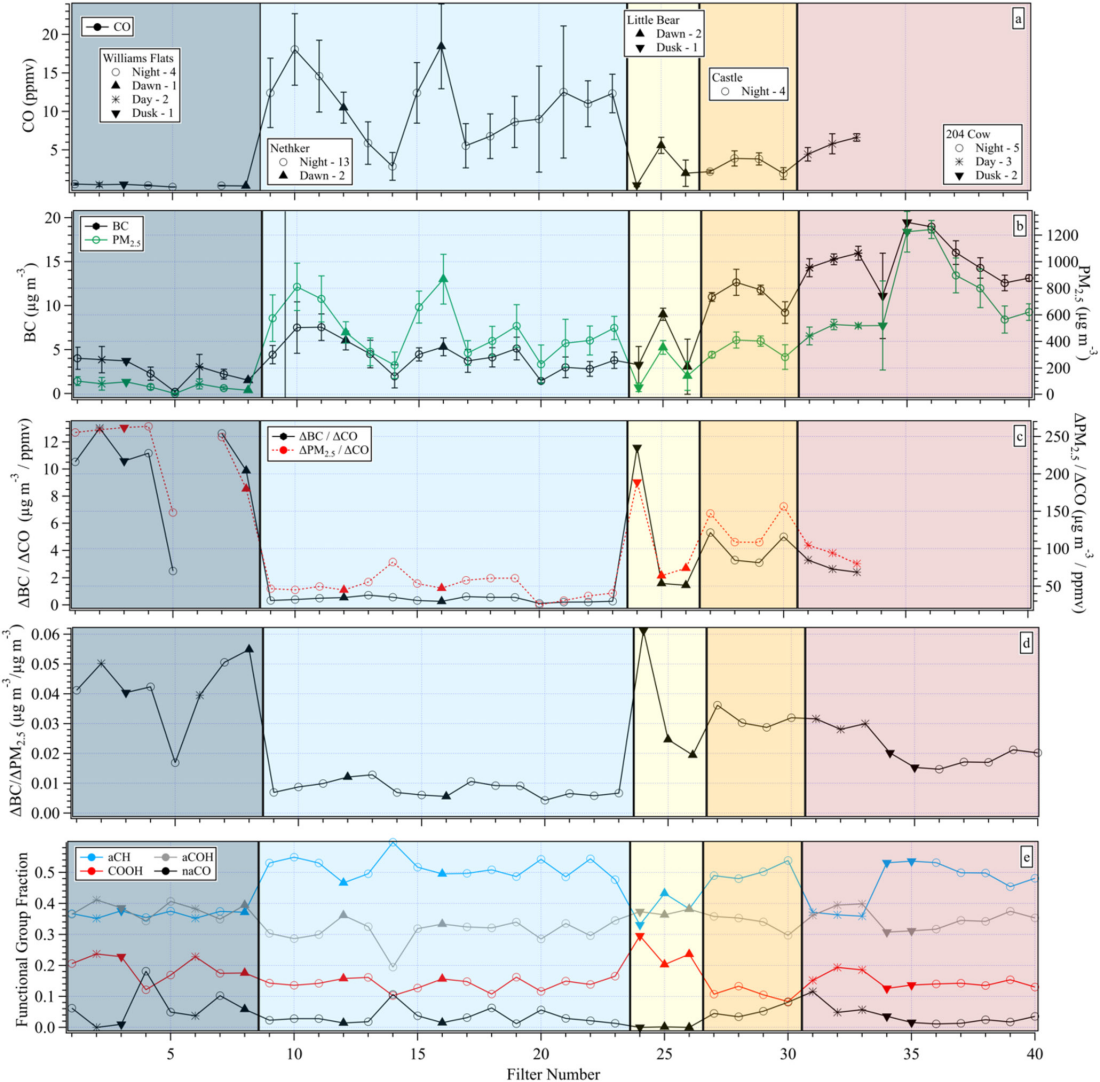
Filter series with different markers (all panels) that denote whether samples were collected at night (circles), dawn (upward triangles), daytime (asterisks), or dusk (downward triangles) with shading to identify fires. (a) CO (ppmv), (b) BC (left) and PM_2.5_ (right) (μg m^−3^), (c) ΔBC/ΔCO (left) and ΔPM_2.5_/ΔCO (right) (μg m^−3^/ppmv), (d) ΔBC/ΔPM_2.5_ (μg m^−3^/μg m^−3^), and (e) fractional contribution of each of the four FT‐IR groups to their sum: aliphatic CH (aCH), alcohol COH (aCOH), carboxylic acid (COOH), and non‐acid carbonyl (naCO). Note, ΔBC, ΔCO, and ΔPM_2.5_ for FN‐5 are all <0 (see Table S3 in Supporting Information S1), that is, background air, even though the ratios of these terms for this sample are not.

**Figure 3:**
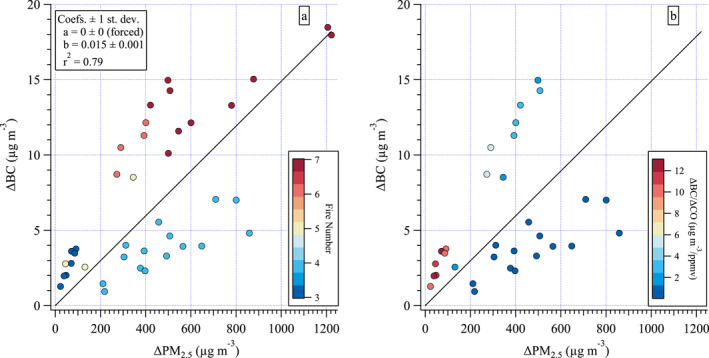
ΔBC versus ΔPM_2.5_ for all filters colored by (a) fire number, and (b) ΔBC/ΔCO (μg m^−3^/ppmv). FN‐5 not shown as ΔBC and ΔPM_2.5_ are both <0 (Table S3 in Supporting Information S1). Missing data points in panel (b) compared to panel (a) are due to missing CO data. Line fit to all data shown in black.

The series of filter mean observations of CO, BC, and PM_2.5_ are shown in Figure 2, along with ΔBC/ΔCO, ΔPM_2.5_/ΔCO, ΔBC/ΔPM_2.5_, and the fraction of the four functional groups measured using FT‐IR. Background colors are used to distinguish the samples from the five fires. Unfortunately, the CO instrument overheated at the 204 Cow fire and did not record reliable data for any of the seven filters collected on the second day. A similar problem occurred during Williams Flats, but only affected one filter sample (FN‐6, Table 1 and Figure 2a). This is a significant limitation affecting 20% of the 40 filter samples. Fortunately, ΔBC/ΔPM_2.5_ captures differences in aerosols across the fires consistent with ΔBC/ΔCO (Figures 2 and 3). The values of ΔBC/ΔCO tend to differ from one fire to the next (Figures 2c and 3) with ΔBC/ΔPM_2.5_ (Figures 2d and 3) also capturing the shift from low (Nethker) to high (Williams Flats) ratios. Given the gaps in CO, we use ΔBC/ΔPM_2.5_ to assess the relative contributions of flaming and smoldering to the sampled smoke. Comparing the filter series of ΔBC/ΔCO and ΔBC/ΔPM_2.5_ (Figures 2c and 2d) and relationships shown in Figure 3, this is not an unreasonable approximation, although we acknowledge some degree of atmospheric processing also contributes to the ΔBC/ΔPM_2.5_ ratio.

#### 3.1.4 FT‐IR Functional Group Composition

Differences between the fires were also observed in the FT‐IR functional group fractions (Figure 2e). Williams Flats, Little Bear, and the first day of sampling at 204 Cow (the first 3 samples at that fire) all tended to exhibit comparable fractions of aliphatic CH (aCH) and alcohol COH (aCOH) groups. In contrast, aCH was the dominant group (∼50% of functional groups) for Nethker, Castle, and the second day of 204 Cow with a reduced fraction of aCOH. This might suggest compositional differences arising as a function of fire intensity given the relatively high values of ΔBC/ΔCO and ΔBC/ΔPM_2.5_ for Williams Flats, low values for Nethker and moderate values for the remainder (other than the first Little Bear sample FN‐24). But correlations between these fractions and ΔBC/ΔPM_2.5_ are only moderate (r^2^ ∼ 0.6 for aCH) to weak (∼0.3 for aCOH and COOH) to uncorrelated (∼0.06 for naCO). This lack of correlation is evident when comparing the composition between Castle and the first three samples from 204 Cow, all of which have very similar values of ΔBC/ΔPM_2.5_ (Figure 2d), yet the composition differs. Further, Little Bear has one high and two moderate ΔBC/ΔPM_2.5_ values, yet limited variability in composition across the functional groups. This suggests the difference in composition is not fully captured by the relative contribution of flaming and smoldering, whether the ΔBC/ΔCO or ΔBC/ΔPM_2.5_ metric is used.

#### 3.1.5 FN‐5 and FN‐24

Overall ΔBC/ΔPM_2.5_ tended to be relatively consistent for all samples from a given fire. There are two exceptions to this. As noted above, FN‐5 from Williams Flats is the only sample collected in predominantly background air rather than a smoke plume (see Table S3 in Supporting Information S1). FN‐24 exhibits a higher degree of flaming than the other two samples at Little Bear likely due to sampling location and time of day. In Figure 2 different symbols are used to differentiate samples collected during nighttime, dawn, daylight, and dusk hours. Dawn and dusk are approximately 6–9 a.m. or p.m., local time, respectively (see Table S1 in Supporting Information S1 for exact sampling times). Most filter samples were collected at night (26). Only 5 daytime samples (all in the afternoon) were acquired: 2 at Williams Flats and 3 the first day at 204 Cow. It can be challenging for a ground‐based mobile laboratory to sample flaming smoke because it is efficiently lofted vertically in daylight hours, whereas at night it can become trapped in the nocturnal boundary layer allowing such emissions to be sampled along with those from smoldering (Burling et al., 2011; Selimovic et al., 2019). At Little Bear, MACH‐2 had a rare opportunity to sample at an elevated site due to the local terrain at dusk (FN‐24) capturing a more flaming plume than was observed anywhere other than Williams Flats (Figures 2c and 2d). Returning to the same elevated site at dawn the following morning (FN‐26) MACH‐2 observed more smoldering smoke as would be expected for an overnight fire.

### 3.2 Measured Optical Spectra With Calculated Single Scattering Albedo (*ω*) Spectra

**Figure 4:**
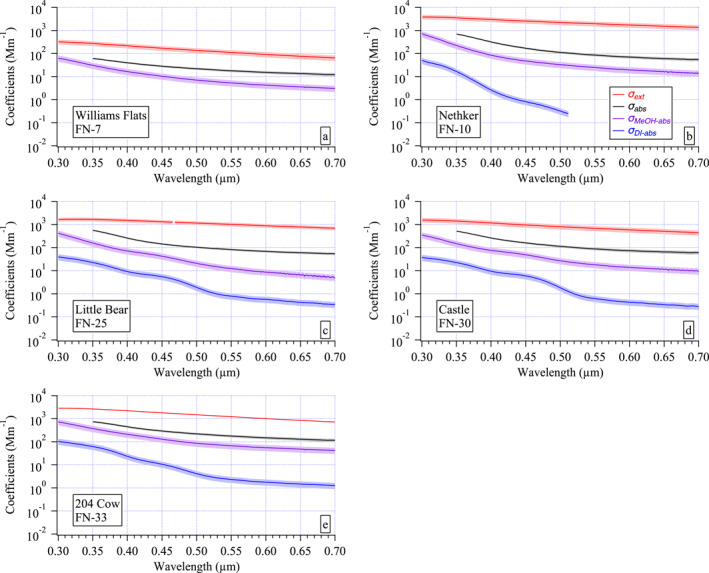
Examples of the measured filter spectra sets for each fire: (a) Williams Flats, (b) Nethker, (c) Little Bear, (d) Castle, and (e) 204 Cow. Mean *σ*_ext_(0.30–0.70 μm) ± 1 standard deviation represent the ambient aerosol and its variability over the filter sampling period (red curves). The shaded errors for all absorption coefficient spectra were derived from uncertainty propagation: *σ*_abs_(0.35–0.70 μm), black, *σ*_MeOH‐abs_(0.30–0.70 μm), purple, and *σ*_DI‐abs_(0.30–0.70 μm), blue. Note that the six orders of magnitude shown on the *y*‐axes render some of the uncertainty shading difficult to discern at this scale. The individual filter number (see Table 1 and Table S1 in Supporting Information S1) from each fire is shown in the label of each panel.

To illustrate some of the differences observed in the optical spectral data between the fires described above, one sample spectra set from each fire is shown in Figure 4. These differences include differing magnitudes in the optical coefficients, curvature of the spectra, spectral features, and the wavelength range of measurable soluble chromophores. An example of the differing magnitudes arises from the comparison of the set from Williams Flats to the other four fires (Figure 4). Although Williams Flats was the most intense fire, MACH‐2 was furthest downwind from this fire (Table 1) such that at the surface the dilute smoke plume exhibited smaller coefficients than observed at the other fires with lower intensity sampled nearer to the source.

**Figure 5:**
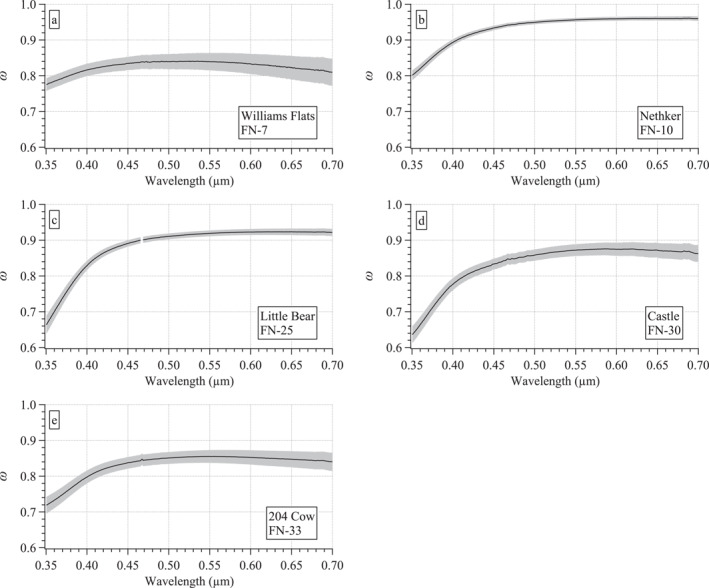
Examples of calculated *ω*(0.35–0.70 μm) spectra for each fire: (a) Williams Flats, (b) Nethker, (c) Little Bear, (d) Castle, and (e) 204 Cow. These examples are calculated from the *σ*_ext_ and *σ*_abs_ spectra shown in Figure 4. Shaded errors estimated from uncertainty propagation.

Differences in the curvature of the *σ*_abs_(*λ*) spectra were evident among the fires, with a flatter spectrum in the Williams Flats example compared to those of Nethker or Little Bear (Figure 4). This contrast is more easily seen in the calculated single scattering albedo spectrum (*ω*(*λ*) = (*σ*_ext_(*λ*) − *σ*_abs_(*λ*))/*σ*_ext_(*λ*)) that is relatively flat for Williams Flats compared to the other fires and exhibits the lowest values throughout the visible portion of the spectrum (Figure 5). The smoldering Nethker fire exhibits the highest *ω* values across the spectral range compared to the other fires with greater curvature than the Williams Flats example. The lowest *ω* values in these examples were found at 0.35 μm for Castle (∼0.64) and Little Bear (∼0.66). These last two examples also exhibit the greatest curvature of the five *ω*(*λ*) spectra shown.

Since *ω* is often used as a proxy for differences in aerosol composition, these spectra suggest differing aerosol composition between fires consistent with differences shown in the previous section for ΔBC/ΔPM_2.5_ and the functional group fractions (Figures 2d and 2e). The *ω*(*λ*) spectral data reported here are novel. Few such spectra exist in the literature (e.g., Jordan, Stauffer, Lamb, Novak, et al., 2021) over this spectral range (0.35–0.7 μm) and hyperspectral resolution (∼0.0008 μm). The differences observed between fires here suggest that wider adoption of this kind of measurement approach may prove useful to better discriminate fires with varying properties (e.g., fuels consumed, fire state and intensity, atmospheric processing as described in Section 2.1) by examining the differences manifested in their *ω*(*λ*) spectra. Further, calculations of direct radiative forcing and satellite retrievals of aerosol optical thickness are sensitive to *ω*(*λ*) (e.g., Reid et al., 1999). Hence, expanding measurements of ambient in situ hyperspectral *ω*(*λ*) would be beneficial in constraining such calculations over a broader range of aerosols found in the ambient atmosphere.

In addition to varying curvature across the spectra sets shown in Figures 4 and 5, differences in spectral features (i.e., subtle broad peaks) in the soluble absorption spectra (Figure 4) were also observed. The *σ*_MeOH‐abs_ spectra exhibit such a feature between 0.4 and 0.5 μm in the Little Bear and Castle examples (Figures 4c and 4d) that was not evident in the Williams Flats, Nethker, or 204 Cow examples (Figures 4a, 4b and 4e). Similar features were even more prominent in the *σ*_DI‐abs_ spectra for all fires for which there is data (Figures 4b–4e).

Finally, note that several *σ*_DI‐abs_ spectra measured at Nethker and 204 Cow were partial (i.e., the measurement was below detection at longer wavelengths, e.g., the Nethker sample in Figure 4b). In contrast, *σ*_MeOH‐abs_ absorption spectra were always above detection over the full wavelength range, typically 2–3 orders of magnitude above detection at 0.7 μm. As a reminder, the truncated *σ*_abs_ measurement range (0.35–0.70 μm) was due to an instrument issue (see Section 2.4) not a limit of detection as was the case for the *σ*_DI‐abs_ spectra.

### 3.3 Characterizing Spectral Variability via Spectral Fitting

The spectra sets vary between fires and from sample to sample. Curve fitting is typically applied to spectra to characterize spectral shape and variability with a tractable parameter or two that can be used to quantify the observed differences. As discussed in Section 1, *α* provides a single parameter used to describe spectral differences, whether in reference to absorption (i.e., distinguishing BC from BrC or dust) or in reference to extinction/scattering (i.e., distinguishing small from large particle populations). The two parameters (*a*_1_, *a*_2_) also described in Section 1 can be used in a similar way, while also accounting for curvature.

For example, spectral curvature in LN(*σ*_ext_) has been shown to depend primarily on the accumulation mode size distribution (both mode radius and the geometric standard deviation, GSD, of the lognormal size distribution) with a minor dependence on composition, that is, the fraction of BC in the aerosol (Eck, Holben, Ward, et al., 2001; Reid et al., 1999). Very small particles (mode radius <100 nm) exhibit nearly linear spectra. Curvature becomes increasingly negative as particle radius increases throughout the accumulation mode (e.g., Eck, Holben, Ward, et al., 2001). For accumulation mode particles, as GSD becomes narrower, curvature becomes more negative (e.g., Eck, Holben, Ward, et al., 2001). Positive spectral curvature is also possible in bimodal size distributions as a function of the coarse mode (Schuster et al., 2006). Here, biomass burning aerosols (BBAs) are dominated by the accumulation mode. Further, the filter samples were collected behind a PM_2.5_ cyclone limiting any coarse fraction contribution to the measured particles. Hence, positive curvature is not expected in this data set.

**Figure 6:**
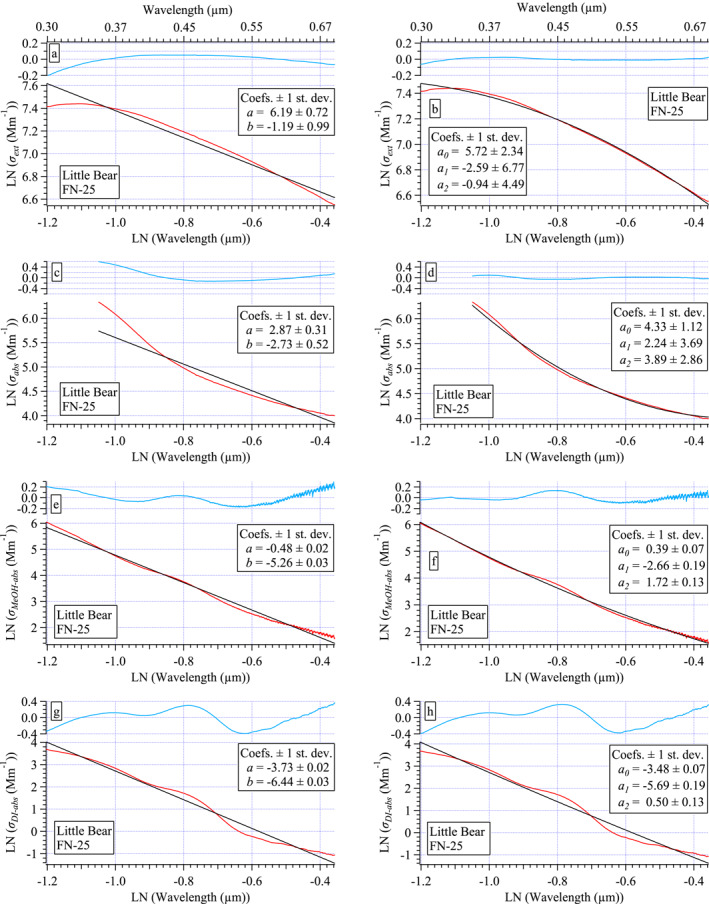
Illustration of linear fits (black curves, left panels with slope, *b*, and intercept, *a*) and second‐order polynomial fits (black curves, right panels, with second‐order coefficient *a*_2_, linear coefficient *a*_1_, and intercept, *a*_0_) compared to measured spectra (red curves, all panels) with residuals (blue curves, = measured—fit spectrum) shown for *σ*_ext_ (a and b), *σ*_abs_ (c and d), *σ*_MeOH‐abs_ (e and f), and *σ*_DI‐abs_ (g and h). All spectra from the Little Bear FN‐25 sample. The *x*‐axis labels in wavelength units of μm shown along top panels for convenience.

Jordan, Stauffer, Lamb, Hudgins, et al. (2021) and Jordan, Stauffer, Lamb, Novak, et al. (2021) made similar in situ aerosol measurements during the KORUS‐OC field campaign in 2016 to those presented here and found spectral curvature was prevalent in all spectra, absorption as well as extinction. In that work, following Schuster et al. (2006), second‐order polynomials were used to improve the fit to the measured spectra. Here again, the quality of second‐order polynomial fits to the measured spectra is compared to that of linear fits. Using the Little Bear sample FN‐25 as an example in Figure 6, each measured and logarithmically transformed spectrum (red curves, all panels) are fit either linearly (black curves, left panels) or with a second‐order polynomial (black curves, right panels). Note, the wavelengths used in the logarithmically transformed spectra for the fits must be in units of μm (see Jordan, Stauffer, Lamb, Hudgins, et al., 2021 for details). The difference between the measured spectrum and the fit spectrum is the residual of the fit (blue curves above each fit, Figure 6). If there is curvature in the residual, it is revealing structure in the measurement that the mathematical function fails to capture. Histograms of the fit residuals from the entire data set show that second‐order polynomials provide a better fit to the data (see Figure S10 in Section S6 in Supporting Information S1).

The improvement in the fit to the *σ*_DI‐abs_ is more modest than for the other spectra sets (Figure S10 in Supporting Information S1). This result contrasts with that of the KORUS‐OC data set, where the improvement reflected by the histogram distributions for *σ*_DI‐abs_ was similar to those shown here in Figure S10 in Supporting Information S1 for the other three spectra sets. The difference in the two studies arises from the spectral features evident in the biomass burning samples in this study (Figure 4). These features appear throughout the spectral range and are not well fit by either a line or second‐order polynomial (Figures 6g and 6h). The implications of this will be discussed further in Section 4.

It is interesting to note that the spectral feature evident at ∼0.45 μm (LN(0.45) = −0.8) in both *σ*_DI‐abs_ and *σ*_MeOH‐abs_ in Figure 6 does not appear in *σ*_abs_. Similar observations have been reported previously (e.g., Cheng et al., 2016; Hinrichs et al., 2016; Lin et al., 2017). Lin et al. (2017) attributed this to a solvent effect, specifically a change in pH between the aerosol and solution phase that resulted in a red‐shift in *σ*_DI‐abs_ compared to *σ*_abs_ thereby underestimating aerosol‐phase UV absorption and overestimating visible absorption. Hinrichs et al. (2016) also attribute this to pH and noted that it might occur in aerosol phase for chromophores adsorbed onto mineral aerosol substrates. We also observed a similar solvent effect (a shift in the wavelength and amplitude of spectral features) evident in laboratory tests using nigrosine aerosol nebulized from a DI solution for tests of the *σ*_abs_ measurement versus in the bulk solution for tests of the LWCC measurement system (see Figure S11 in Section S7 in Supporting Information S1). Here, pH was not measured either for the ambient samples (Figures 4 and 6) or for the nigrosine tests (Figure S11 in Supporting Information S1), hence the solvent effect that led to the observed spectral shifts cannot be confirmed. These observations illustrate the need to measure hyperspectral *σ*_abs_ to accurately represent the spectral shape of ambient in situ aerosols rather than reconstruct the spectral shape from bulk solution phase measurements (as described in Section 2.3). Ideally this would be accomplished with a time‐of‐flight in situ measurement technique, but to our knowledge no such instrument exists that can measure hyperspectral information from 0.3 to 0.7 μm at ambient concentrations. We hypothesize that the filter based *σ*_abs_ measurement here more closely resembles that which would be obtained from a time‐of‐flight approach, but more research is needed to establish whether that assumption is correct. It is possible filter effects may also lead to spectral changes from an ambient time‐of‐flight measurement.

### 3.4 Linear and Second‐Order Polynomial Fit Parameters: Their Relationship and Interpretation

**Figure 7:**
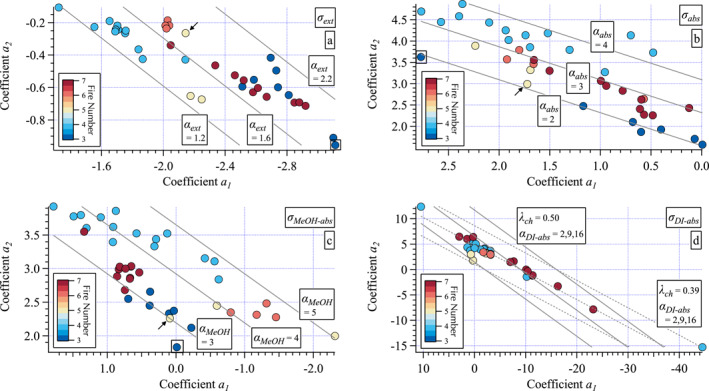
Maps of (*a*_1_, *a*_2_) for all measured spectra: (a) *σ*_ext_, (b) *σ*_abs_, (c) *σ*_MeOH‐abs_, and (d) *σ*_DI‐abs_. All panels show (*a*_1_, *a*_2_) calculated from fits over LN(0.35–0.70 μm) to enable direct comparison across all panels. Data points colored by fire number (see enumeration in Table 1 or color code in Figure 1) with lines of constant *α* (gray) calculated using the mean *λ*_ch_ for each set (∼0.51 μm for all but *σ*_DI‐abs_ where *λ*_ch_ ranges from ∼0.39 to ∼0.50 μm, see text). The first Little Bear sample (FN‐24) is highlighted with an arrow and the predominantly background air sample from Williams Flats (FN‐5) is enclosed in a box (panels (a–c) only).

Each data point shown in Figure 7 represents the (*a*_1_, *a*_2_) coefficients for a measured spectrum in the *σ*_ext_, *σ*_abs_, *σ*_MeOH‐abs_, and *σ*_DI‐abs_ sets. As explained in Section 1, a single value for *α* maps into a line in (*a*_1_, *a*_2_) space with a slope of −2LN(*λ*_ch_), where *λ*_ch_ is approximately constant for any given wavelength range (Equation 4). *λ*_ch_ for each measured spectrum is calculated from the fit parameters *α* and (*a*_1_, *a*_2_). The mean ± standard deviation of *λ*_ch_ for the four sets are 0.508 ± 0.002 μm (*σ*_ext_), 0.524 ± 0.020 μm (*σ*_abs_), 0.504 ± 1.3e−8 μm (*σ*_MeOH‐abs_), and 0.486 ± 0.031 μm (*σ*_DI‐abs_). With the exception of *σ*_DI‐abs_, fits over the LN(0.35–0.7 μm) range result in *λ*_ch_ ∼0.51 μm for all sets with somewhat greater variability found for the *σ*_abs_ set. The exception of *σ*_DI‐abs_ here arises from partial spectra (e.g., Figure 4b), that is, some portion of the spectrum is below detection. A partial spectrum results in a reduction from the nominal value expected for the full wavelength range. This results in skewed mapping in (*a*_1_, *a*_2_) space as shown in Figure 7d. See Section S8 in Supporting Information S1, Jordan, Stauffer, Lamb, Hudgins, et al. (2021) and Jordan, Stauffer, Lamb, Novak, et al. (2021) for further details. Note, where there were complete spectra for *σ*_DI‐abs_ the mean and standard deviation were the same as for *σ*_MeOH‐abs_.

Lines of constant *α* are shown along with (*a*_1_, *a*_2_) for each spectrum for all four measured spectra sets (Figure 7), where points with similar values of *α* are oriented in lines from the upper left to lower right in (*a*_1_, *a*_2_). The (*a*_1_, *a*_2_) map provides separation in terms of curvature (*a*_2_) and the axis of symmetry (= −*a*_1_/2*a*_2_, represented by separation along the *a*_1_ axis, see Section S8 in Supporting Information S1 for details) for the parabola described by the second‐order polynomial. Hence, it is possible to examine drivers of the difference across (*a*_1_, *a*_2_) space for any given value of *α* (e.g., consider *α*_MeOH‐abs_ = 4.0 with different (*a*_1_, *a*_2_) values observed from Nethker and Little Bear/Castle samples, Figure 7c). Note, the large range in values of (*a*_1_, *a*_2_) found for *σ*_DI‐abs_ arise from the partial spectra measured (which have smaller values of *λ*_ch_ as illustrated in Figure 7d) and hence, lead to a different distribution in (*a*_1_, *a*_2_) space than the other three sets (see Section S8 in Supporting Information S1 for further details). Given the absence of data from Williams Flats, along with the complications arising from the partial spectra, *σ*_DI‐abs_ will be set aside for a future study. Here, the rest of the discussion will focus solely on the three sets for which we have complete spectra for all five fires.

The striking result evident in Figure 7 is that the coefficients of spectra from a given fire tend to cluster together in (*a*_1_, *a*_2_) space. This suggests that second‐order polynomials contain additional information on the differing aerosol characteristics across fires than what *α* alone can provide. The three most likely avenues to explore are those described in preceding sections: biofuels consumed (due to differing ecosystems and/or fire state), fire state (combustion phase and completeness of combustion), and atmospheric processing (partially parameterized by distance downwind of sampling from emission).

The negative curvature of LN(*σ*_ext_) means that its greatest curvature (i.e., greatest absolute value of *a*_2_) from this data set is found from the Williams Flats (#3), 204 Cow (#7), and two of the Little Bear (#5) spectra (Figure 7a). The least curvature in LN(*σ*_ext_) was found in the Nethker (#4) and Castle (#6) spectra. The four Castle samples were collected over 4 hr one night, each sample for ∼1 hr (Table S1 in Supporting Information S1). This set of samples exhibited little variability in (*a*_1_, *a*_2_) or *α*_ext_ compared to the samples collected over a span of 14 hr at Little Bear to multiple days at the other sites. The range of values for *α*_ext_ (∼1.2 to 2.2) might suggest that the size distributions of these samples are intermediate between large particles (*α*_ext_ ≤1, e.g., sea salt and dust) and small particles (*α*_ext_ ≥2, e.g., combustion) (Schuster et al., 2006). However, the values of *α*_ext_ depend on the wavelengths used to calculate them. The wavelength range used to calculate *α*_ext_ in Figure 7a (LN(0.35–0.7 μm)) results in smaller values than would be found from a narrower visible range such as LN(0.45–0.632 μm), see also Jordan, Stauffer, Lamb, Hudgins, et al. (2021). Hence, caution in the interpretation of the values in Figure 7a (due to the *λ*_ch_ dependence of the values) is advised. As noted in Section 3.3, all the aerosols in this study are in the PM_2.5_ fraction, dominated by the accumulation mode in biomass burning emissions. Extinction spectra are driven predominantly by aerosol scattering and hence, size distribution. A future study is planned to use Mie theory, constrained by the measured *σ*_abs_ spectra, to examine the sensitivity of the measured *σ*_ext_ to BrC. The remainder of this work focuses on the absorption spectra, a necessary first step prior to the Mie theory study.

**Figure 8:**
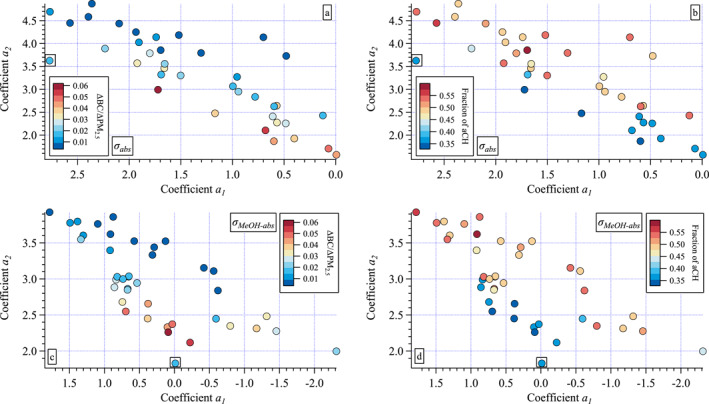
Maps of (*a*_1_ *, a*_2_) for ΔBC/ΔPM_2.5_ (a and c) and the fraction of aCH (b and d) for *σ*_abs_ (a and b) and *σ*_MeOH‐abs_ (c and d). All panels show (*a*_1_, *a*_2_) calculated from fits over LN(0.35–0.70 μm) to enable comparison between the two sets of absorption spectra. The point enclosed in a box in all panels highlights the predominantly background air sample from Williams Flats (FN‐5). Note, compare to Figures 7b and 7c to relate data points here to specific fires.

For *σ*_abs_, the clustering of (*a*_1_, *a*_2_) points for each fire (Figure 7b) resembles the parallel line arrangement of *α*_abs_ (Figure 7c) where the bottom edge of the distribution (smallest *α*_abs_) includes the samples with the largest fraction of ΔBC/ΔPM_2.5_ (Figure 2d), that is, the first Little Bear sample and most of the Williams Flats samples. The one sample collected predominantly in background air outside of a smoke plume (FN‐5 from Williams Flats) is highlighted with a box in Figure 7b. It has the same value of *α*_abs_ but is not otherwise associated with the rest of the Willams Flats points in this set. FN‐5 nominally has a low value of ΔBC/ΔPM_2.5_ but it is derived from negative values of ΔBC and ΔPM_2.5_, and hence parameterizing this sample by ΔBC/ΔPM_2.5_ is not valid by definition. The Nethker data points define the upper edge of the distribution (largest *α*_abs_)with Castle, 204 Cow, and the remaining two Little Bear points in between these and the lower edge group, as might be expected from ΔBC/ΔPM_2.5_ (Figure 2d). The implication of this is that ΔBC dominates the spectral characteristics of *σ*_abs_ in the curvature mapping domain as it does in the single parameter space of *α*_abs_. Coloring the (*a*_1_, *a*_2_) points for *σ*_abs_ by ΔBC/ΔPM_2.5_ (Figure 8a) is consistent with this interpretation, although the progression across the color scheme is more variable than for *α*_abs_. This will be discussed further in Section 3.5.

The clustering of *σ*_MeOH‐abs_ points for each fire (Figure 7c) suggests that the curvature increases from Williams Flats to 204 Cow to Nethker for the northern fires, which may be due to a shift from intense flaming to smoldering (i.e., a function of ΔBC/ΔPM_2.5_, Figure 8c), but this is confounded by the points from the southern fires. The most flaming sample from Little Bear is among the Williams Flats samples (Figure 7c), while the other two are most closely associated with the Castle samples in an area of the (*a*_1_, *a*_2_) map unoccupied by data from other fires. This cluster of southern fire data points represent values for the axis of symmetry from the parabolic fit found at the longest wavelengths for this set, ranging from ∼1.13 to 1.79 μm. The smallest value of the axis of symmetry 0.80 μm is found for the Nethker sample at the upper left of Figure 7c. This range in the axis of symmetry suggests that the spectral shape is sensitive to curvature along the full measured range. This is consistent with the observation that contributions to *σ*_MeOH‐abs_ were well above detection at *λ* = 0.7 μm. BrC absorption provided by soluble organic compounds is typically assumed to be limited to a wavelength range extending from the UV into the short‐, perhaps, mid‐visible range. However, the results here suggest that organic chromophores contribute throughout the entire visible range and influence the observed spectral curvature.

The clear relationship between *α* and (*a*_1_, *a*_2_) for each optical parameter (Figure 7) is due to the well‐defined mathematical relationship between the linear fit and the parabolic fit. However, as noted in Section 1 both fits are empirical and not derived from first principles of underlying physical and chemical characteristics of the aerosols. The separation across fires found in the (*a*_1_, *a*_2_) maps hints that there are differences in these underlying aerosol characteristics from each fire driving the observed spectral differences. The challenge then is to identify an approach that can be used to relate the optical differences back to the key underlying aerosol drivers of those differences. An initial test of this idea will be presented in Section 3.5 with a more complete discussion of the problem presented in Section 4.

### 3.5 Mapping the FT‐IR Functional Groups Into (*a*_1_, *a*_2_) Space

One approach to identify relationships between aerosol characteristics and curvature is to map other individually measured or derived parameters into (*a*_1_, *a*_2_) space. However, this did not prove to be very informative. All of the ancillary measurements were examined in this way, but only the two best examples providing some insight are shown here (Figure 8). In addition to the mapping of ΔBC/ΔPM_2.5_ in (*a*_1_, *a*_2_) for *σ*_abs_ (Figure 8a) described above in Section 3.4, the fraction of each of the four FT‐IR functional groups were also mapped. Three of the four groups are oxygenated, but the fourth (aCH) represents aliphatic CH groups (i.e., alkanes). Hence, the fraction of aCH compared to the other three groups provides an indication of more or less oxidation of the organic aerosol. As was found for ΔBC/ΔPM_2.5_ (Figure 8a), the fraction of aCH broadly separates across the distribution (small to large *α*_abs_ as in Figure 7b) in the *σ*_abs_ (*a*_1_, *a*_2_) map (Figure 8b) such that the smallest fraction of aCH occurs in aerosols with the largest ΔBC/ΔPM_2.5_ ratio. This could be due to preferential aCH production from smoldering, or it could be related to increased oxidation with distance downwind from the source. However, as noted in Section 3.1, aCH is only moderately anticorrelated (slope = −3.64 ± 0.50) to ΔBC/ΔPM_2.5_ with an *r* ^2^ = 0.59 and there is no clear progression of the observed values across the (*a*_1_, *a*_2_) space.

The same behavior is found for ΔBC/ΔPM_2.5_ and fraction of aCH mapped into the *σ*_MeOH‐abs_ (*a*_1_, *a*_2_) parameter space (Figures 8c and 8d). There is separation between the high and low values, but the mapping of the range of values is imprecise suggesting the spectral shape of *σ*_MeOH‐abs_ is not that sensitive to these individual parameters. As will be discussed in Section 4, light absorption depends on specific molecular structures within the aerosol (chromophores). The FT‐IR measurements provide information on the relative amounts of the four groups measured but cannot distinguish those within chromophoric structures from the rest. Further, important chromophores such as conjugated *π* bonds and substituted heteromolecules such as nitrogen were not measured. As will be discussed further in Section 4 the aerosol chromophores within any given smoke plume are expected to be found within a wide variety of molecules in complex mixtures deriving from a range of fire intensities consuming diverse biofuels. This suggests that a simple mapping strategy of individual parameters as in Figure 8 is unlikely to provide strong correlations to (*a*_1_, *a*_2_). Instead (*a*_1_, *a*_2_) should be viewed as a parameterization of a complex suite of factors that contribute to observed aerosol optical properties. Finally, even (*a*_1_, *a*_2_) is a simplification of the measured spectra. Hyperspectral measurements and more complex mathematical approaches are expected to be more sensitive to the drivers of aerosol optical variability.

## 4 Discussion

This section provides a synthesis of recent observations both in the laboratory and in the ambient environment of BBOA and various contributions to their absorption spectra. These observations are best understood in the context of how fire produces these aerosols. Simple parameterizations, whether *α* or (*a*_1_, *a*_2_), cannot fully capture the observed spectral complexity, prompting the question of how else might hyperspectral observations be used to inform model parameterizations and scientific understanding.

### 4.1 Synthesis of Current Understanding of BBOA Chromophores

#### 4.1.1 Aerosol Chromophoric Structures and Recent Laboratory Observations of BBOA

The graphitic sheets of BC are very efficient absorbers all across the UV‐visible‐IR wavelengths of light (e.g., Bond et al., 2013; Desyaterik et al., 2013; Yang et al., 2009), whereas the wavelength dependence of organic BrC molecules depend on the number and structure of conjugated (*π*) bonds, functional groups, and heteroatoms, especially N (e.g., Andreae & Gelencsér, 2006; Apicella et al., 2004; Bones et al., 2010; Chen & Bond, 2010; Desyaterik et al., 2013; Jacobson, 1999; Moosmüller et al., 2009; Updyke et al., 2012). Other organic molecular structures do not absorb light. Common moieties found in pigments suggest the kinds of structures that may absorb light in BrC; these include quinones, terpenoids, flavonoids, and porphyrins (e.g., chlorin, a reduced form of a porphyrin, is the ring structure within chlorophyll) (Schaller, 2020). Similar moieties and their fragments (e.g., pyrrole and pyridine heterocyclics found in porphyrins) are observed in BrC chromophores (Fleming et al., 2020; Roberts et al., 2020).

The 2016 NOAA FIREX conducted at the U.S. Forest Service Fire Science Laboratory in Missoula, MT provided comprehensive measurements of 34 different biomass fuels common in the western U.S. burned in 75 stack burns (Fleming et al., 2020; Jen et al., 2019; Roberts et al., 2020; Selimovic et al., 2018). Jen et al. (2019) separated a total of ∼3,000 unique organic compounds from aerosols analyzed from 29 of the burns. Of those, only 400 could be classified into broad categories with 149 identified as specific compounds; the remainder could not be classified. From any given stack burn, a range of 97–834 organic compounds were separated with the classified compounds accounting for 4%–37% of the total organic mass (mean 20% ± 9%). The 11 classifications were non‐cyclic aliphatic/oxygenated, sugars, PAHs (methylated and oxygenated), resin acids/diterpenoids, sterols/triterpenoids, organic N, oxygenated aromatic heterocycles, oxygenated cyclic alkanes, methoxyphenols, substituted phenols, and substituted benzoic acids. The separated compounds represent intermediate and semi‐volatile organic compounds. The explicitly identified compounds all had molecular weights (MWt) ≤576 amu, with most ≤400 amu.

The results from Jen et al. (2019) reveal that any given organic compound within the set that comprise BBOA will only contribute a fraction to the total mass, a very small fraction for most. Further, important aerosol chromophores appear to be present in trace amounts compared to non‐absorbing molecules that may dominate aerosol mass (e.g., Bones et al., 2010; Palm et al., 2020; Updyke et al., 2012; Washenfelder et al., 2022). In the same 2016 FIREX study, Fleming et al. (2020) performed a similarly comprehensive examination of the BrC components within the aerosols identifying 46 chromophores (MWt ≤400 amu with above‐detection light absorption limited to *λ* ≤0.55 μm over a measured wavelength range of 0.3–0.7 μm) from a diverse set of biomass samples (from 7 coniferous [gymnosperm] and 3 flowering [angiosperm] species, including duff, litter, and canopy material). The chromophores were grouped into four categories: lignin derived products (including lignin pyrolysis products), distillation products (including coumarins and flavonoids), nitroaromatics, and PAHs. While representatives of these classes were present from most fuel types, specific chromophores varied based on plant type (e.g., gymnosperm vs. angiosperm) and ecosystem components burned (e.g., duffs, litter, and canopy).

#### 4.1.2 Biomass Burning Production of Organic Aerosol Chromophores

##### 4.1.2.1 Production of Aerosols via Flaming/Smoldering Fire Stages, Influenced by Environmental Characteristics

To understand Fleming et al.'s (2020) BrC categories, it is helpful to understand the processes involved in combustion: ignition, flaming, glowing, smoldering, and extinction (Lobert & Warnatz, 1993). The important stages for aerosol generation are flaming and smoldering. Flaming combustion has 2 stages: solid (or condensed) fuel decomposition, and gas (flame) phase. The solid phase decomposition occurs via pyrolysis, smoldering, and glowing combustion with greater organic aerosol emissions (and chemical diversity) from lower temperature processes. The flaming stage involves heterogeneous processes, first elevated temperature induces drying and distillation of the fuel water and volatile compounds that are driven away from the heat either into the bulk fuel material or into the atmosphere. Second, pyrolysis is the temperature driven cracking of the fuel molecules, that is, large MWt compounds are decomposed to smaller MWt compounds. Third, as flammable gases are produced the gas‐phase flaming converts the primary decomposition products to secondary lower MWt products. Drying and distillation commence at relatively low temperatures, pyrolysis starts at ∼400 K and remains endothermic until ∼450 K, above which combustion becomes exothermic and self‐sustaining potentially reaching peak temperatures in the fuel bed up to ∼1800 K. After the flammable gases are exhausted, smoldering can continue until slower pyrolysis reduces the heat and combustion terminates kinetically (Lobert & Warnatz, 1993).

The heterogeneity of these processes in a wildfire suggests that even while there is gas‐phase flaming, trace amounts of larger MWt compounds may escape into the atmosphere along the temperature gradient of the fire moving across the landscape via drying and distillation and smoldering pyrolysis (e.g., Stockwell et al., 2016; Yokelson et al., 1997). However, far more such compounds are emitted under low temperature smoldering pyrolysis than high temperature flaming. Further, external conditions such as topography and winds play a role in the efficiency of the combustion with heading fires (moving with the wind) spreading faster with larger, but less efficient flames versus backing fires (moving into the wind) spreading more slowly and consuming the fuel more completely (Lobert & Warnatz, 1993). Fires moving upslope in hilly/mountainous terrain behave like heading fires, while those moving downslope behave like backing fires. Finally, fuel water content (which can range from 5% to 200% of a plant's dry weight, e.g., dead dry season grasses to fresh needles and leaves) dramatically effects combustion with high moisture content limiting the combustion temperature to smoldering pyrolysis or extinguishing the fire completely (Lobert & Warnatz, 1993).

##### 4.1.2.2 Dependence of Aerosol Composition on Biofuels

The composition of the fuel combustion products derives from the composition of the biomass fuels (Lobert & Warnatz, 1993). Organic compounds in plants fall into three categories: (a) primary metabolites, (b) structural high MWt polymers, and (c) secondary metabolites (Gutiérrez‐Lomelí et al., 2012). Primary metabolites are responsible for the metabolism of the plant and the reproduction of its cells (e.g., nucleic acids, the common amino acids, and sugars). The structural polymers include cellulose, lignin, and proteins. The secondary metabolites are produced by the plant to interact with its environment and can be specifically produced to respond to biotic and abiotic stressors (Gutiérrez‐Lomelí et al., 2012), hence, they are variable within the plant. There are tens of thousands of secondary metabolites that fall into six major groups: (a) polyketides and fatty acids, (b) terpenoids (C_5_H_8_)_n_, and steroids, (c) phenylpropanoids (phenolic acid compounds), (d) alkaloids, (e) specialized amino acids and peptides, and (f) specialized carbohydrates (Gutiérrez‐Lomelí et al., 2012, and references therein). Each plant has its own characteristic set. The secondary metabolites include pigments (e.g., flavonoids), antioxidants (e.g., phenols), and essential oils (e.g., terpenoids).

By mass, cellulose and hemicellulose comprise the majority of a plant's dry mass 50%–75% with another 15%–30% from lignin (Lobert & Warnatz, 1993), hence the large MWt structural polymers provide the dominant biomass fuel consumed by fire. Up to 10% of the dry mass is comprised of minerals, with the remainder the primary and secondary metabolites (Lobert & Warnatz, 1993). Returning to the BrC compound classes identified by Fleming et al. (2020) the lignin derived products are largely produced by pyrolysis as are the PAHs. As Fleming et al. (2020) explain, the PAH chromophores individually tend to be weakly absorbing, but collectively they can comprise a significant fraction of the total BrC light absorption. In contrast, the strongly absorbing nitroaromatics are formed in the smoke plume from reactions of aromatics with NO_x_ produced in the smoke plume via gas‐phase flaming (Roberts et al., 2020). Finally, the 4th group, the distillation products that include coumarins and flavonoids are produced in the low temperature dehydration and distillation stage of combustion from the trace metabolite compounds. In short, different stages of combustion process different parts of the plant biomass generating the wide variety of compounds observed in biomass burning OA with some subset of those compounds able to absorb light.

#### 4.1.3 Organic Chromophores Not Limited to Small Compounds or Short Wavelengths

The compounds identified by Jen et al. (2019) and Fleming et al. (2020) were relatively small compounds most of which had MWt ≤400 amu, all ≤576 amu. However, two recent studies have used size exclusion chromatography to look for absorption from large MWt compounds in soluble extracts of ambient biomass burning samples (Di Lorenzo & Young, 2016) and in a laboratory study (Wong et al., 2017). Di Lorenzo and Young (2016) observed three populations of chromophores at *λ* = 0.3 μm in an aqueous extract of 2 days old boreal BBOA with the absorption front (starting from the largest size) at 10,000 amu with maxima at 2,750, 1,900, and 500 amu. They compared this to the absorption of an aqueous extract of Suwannee River Humic Acid (a typical standard used to represent large MWt humic like substances (HULIS) found in ambient atmospheric aerosols) that had a single population of chromophores with the absorption front at 20,000 amu and a maximum at 4,500 amu. Hence, although the ambient biomass burning BrC possessed large MWt compounds, they appear to be distinct from HULIS. They used 0.3 μm to discuss the differing size distributions, but the full range of absorption spectra spanned ∼0.29 to 0.60 μm and showed differences between the BBOA and HULIS samples throughout the measured wavelength range. Overall, they reported that most of absorption occurred in chromophores with MWt ≥1,000 amu with absorption extending into the visible range.

Such large chromophores are consistent with the findings of Saleh et al. (2014) who attributed the dominant biomass burning BrC chromophores to those in the extremely low volatility organic compound class of the volatility basis set (Donahue et al., 2012). Di Lorenzo and Young (2016) suggest that smaller chromophores are likely to be important close to the source of the fire, but atmospheric oxidation removes them leaving a recalcitrant larger population of chromophores further downwind. This interpretation is consistent with the results of Wong et al. (2017) that photolytic aging resulted in a recalcitrant set of large chromophores that dominated the BrC absorption of BBOA from the two initial populations of chromophores in fresh smoke. Even in the fresh smoke, the larger (MWt = 400–66,000 amu) compounds dominated the absorption compared to the smaller compounds (MWt <400 amu).

Wong et al. (2017) noted that not all combustion products were measured in their experiment as they observed a viscous material from low volatility compounds that accumulated in the tubing of their system. They performed a low temperature pyrolysis experiment using small pieces of dried cherry hardwood at 210°C in an N_2_ atmosphere to simulate smoldering. This low volatility viscous material is intriguing given observations of tarballs (unique BBAs) in the ambient atmosphere (e.g., Adachi et al., 2019; Sedlacek et al., 2018). Tarballs exhibit broad spectral absorption from the UV to near IR (Adachi et al., 2019, and references therein). In fresh smoke, tarball aerosol material has low viscosity that rapidly increases via atmospheric processing in the smoke plume becoming highly viscous and spherical in shape with modal diameters ∼220 nm (*σ* = 1.6). Although atmospheric oxidation reactions appear to be crucial in transforming the material into its highly viscous state, gas‐to‐particle condensation and coagulation do not appear to be important, hence, tarballs are considered processed primary organic aerosols (POAs) rather than secondary (SOA) (Adachi et al., 2019; Sedlacek et al., 2018). This is consistent with observations of low MWt compounds that found only 13% ± 3% of the total BBSOA source was derived from gas‐phase precursors (primarily phenolic vapors) with the remainder due to evaporated BBPOA that was subsequently processed to form BBSOA (Palm et al., 2020).

The increase in viscosity of tarballs is accompanied by increases in N:C and O:C ratios in the amorphous organic material that comprises tarballs (Adachi et al., 2019). This suggests oxidation reactions are important in the transformation of this material but confirming the processes that produce tarballs remains an active area of research (Adachi et al., 2019; Sedlacek et al., 2018). Functional groups observed within tarballs include aromatics, ketonic or phenolic groups, and carboxylic groups (Adachi et al., 2019). These low volatility, high viscosity aerosols are likely to be good candidates to look for large MWt chromophores within their amorphous structure.

Fleming et al. (2020) studied the UV photodegradation of BBAs in the 2016 FIREX experiment described above, finding that while individual chromophores underwent exponential decay, irradiated filter samples of the aerosols did not. The filter aerosols exhibited decreasing absorption for a period, then little change over time was observed indicating a recalcitrant population of chromophores. This result is consistent with Di Lorenzo and Young (2016) and Wong et al. (2017). If tarballs contain the large MWt compounds identified in those studies, then perhaps the reduced molecular diffusion in the high viscosity aerosols contributes to their resistance to the rapid photodegradation observed for individual chromophores as in Fleming et al. (2020).

In addition to the individual contributions from the myriad chromophores within BBOA to their combined absorption spectrum, charge transfer complexes from supramolecular interactions of low MWt molecules have been reported to contribute up to 50% of the observed light absorption from 0.3 to 0.6 μm (Phillips & Smith, 2014). Collectively, the research to date indicate that ambient aerosol absorption spectra are best understood as a superposition of multiple contributions to a given spectrum.

### 4.2 Limits of Simple Parameterizations of Optical Spectra

It is within the context of the synthesis of the preceding section that the limitations of simple parameterizations become evident providing the rationale for making routine hyperspectral measurements of aerosol optical properties.

#### 4.2.1 Errors That Arise From Extrapolating From a Limited Range of Wavelengths

**Figure 9:**
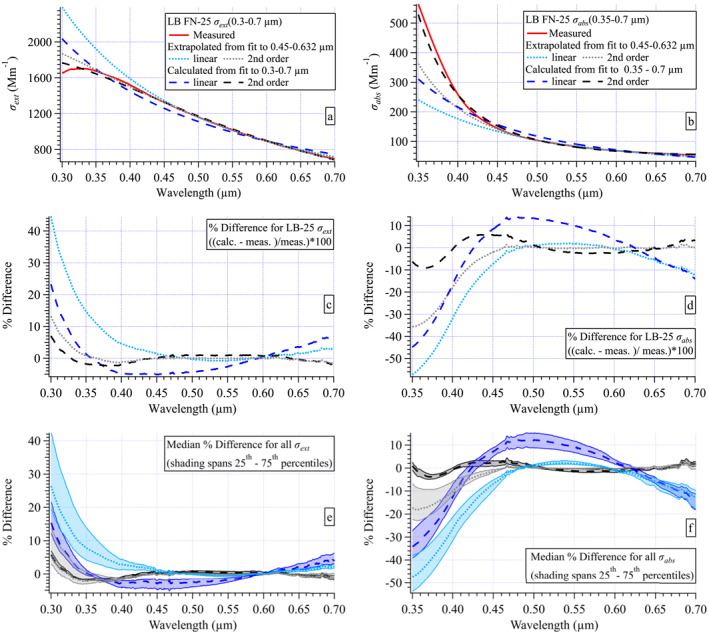
Illustration of measured *σ*_ext_ (a) and *σ*_abs_ (b) in Mm^−1^ units for Little Bear FN‐25, along with calculated spectra from fit coefficients (linear and second order polynomial) over the observed 0.3–0.7 μm range and extrapolated from coefficients of fits to 0.45–0.632 μm range. The percent difference between the calculated (both full range and extrapolated as shown in panels (a and b)) and observed spectra of *σ*_ext_ (c) and *σ*_abs_ (d) for FN‐25. Median percent differences (all filters) of calculated and extrapolated spectra with respect to measured *σ*_ext_ (e) and *σ*_abs_ (f) spectra, shaded to indicate range from 25th to 75th percentiles.

Second order polynomial fits reduce the error (typically within ±10% over the full measured wavelength range, black dashed lines Figure 9) compared to linear fits (typically within ±20%, *σ*_ext_, and ±40% *σ*_abs_, blue dashed lines Figure 9) for parameterized spectra compared to measured *σ*_ext_ and *σ*_abs_. Nonetheless, neither parameterization fully captures the measured spectral shape. Extrapolating across the full measured range from a narrower set of wavelengths (here, 0.45–0.632 μm, typical of wavelengths measured by commercial nephelometers and absorption photometers) leads to much larger errors particularly for linear extrapolations, here typically <40% for *σ*_ext_ and <|55|% for *σ*_abs_ (blue dotted lines Figure 9). Extrapolating second order polynomial fits (gray dotted lines Figure 9) reduces these errors, but they remain larger than fits to the full measured range, particularly for *σ*_abs_ (typically <|22|%, up to |36|% at 0.35 μm, Figure 9d).

**Figure 10:**
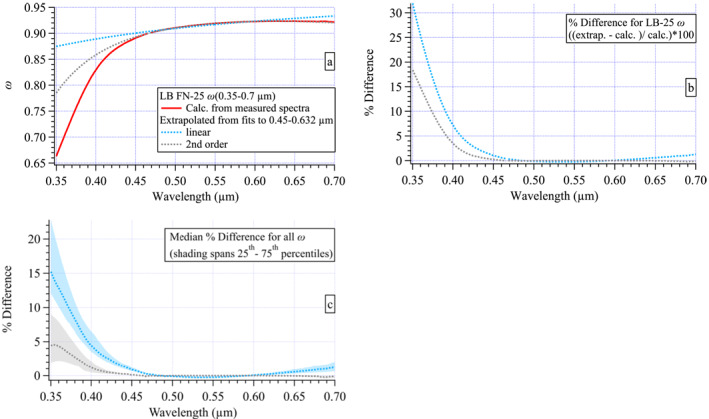
(a) *ω*(0.35–0.7 μm) calculated from measured and extrapolated *σ*_ext_ and *σ*_abs_ spectra shown in Figures 9a and 9b for Little Bear FN‐25. (b) The percent difference between *ω*(0.35–0.7 μm) calculated from the extrapolated spectra and observed spectra of *σ*_ext_ and *σ*_abs_ for FN‐25. (c) Median percent differences (all filters) of *ω*(0.35–0.7 μm) calculated from the extrapolated spectra with respect to measured spectra, shaded to indicate range from 25th to 75th percentiles.

Using the measured and extrapolated spectra shown in Figure 9, *ω*(0.35–0.7 μm) can be calculated and compared (Figure 10). The linear coefficients for Little Bear FN‐25 (*α*_ext_(0.45–0.632 μm) = 1.42 and *α*_abs_(0.45–0.632 μm) = 2.33) lead to ∼32% error in *ω* at 0.35 μm (dotted blue line Figure 10b) compared to that calculated from the measured spectra. The second order coefficients for the 0.45–0.632 μm range ((*a*_1_, *a*_2_)_ext_ = (−2.36, −0.75) and (*a*_1_, *a*_2_)_abs_ = (0.50, 2.30)) reduce this error to 18%. FN‐25 is representative of the 90th percentile percent difference in *ω*(0.35–0.7 μm) found for this data set. At 0.35 μm the median errors among the whole filter set are ∼15% and ∼5% for the linear and second order extrapolations, respectively, with 75th percentile errors up to 23% and 9%.

#### 4.2.2 Spectral Features Not Captured by Simple Parameterizations

**Figure 11:**
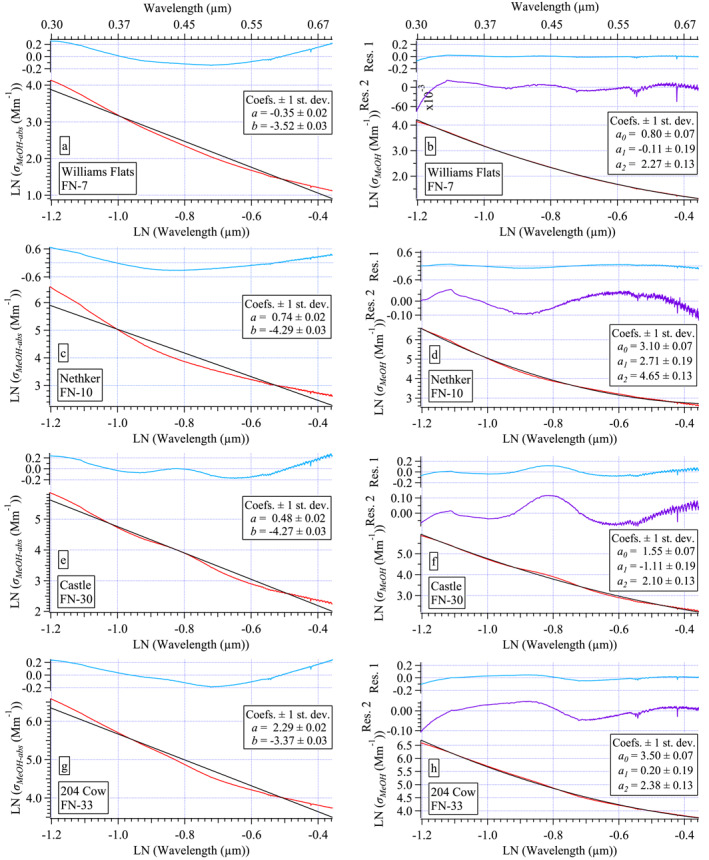
Similar to Figure 6, linear fits (black curves, left panels) and second‐order polynomial fits (black curves, right panels) to measured LN(*σ*_MeOH‐abs_(0.30–0.70 μm)) spectra (red curves, all panels) are shown with residuals at the same scale for both fits (blue curves). The residual fits to the second‐order polynomial fits are rescaled (purple curves, right panels) to highlight spectral features. The examples shown are the same as those in Figures 4 and 5, except for the Little Bear example already provided in Figure 6. The *x*‐axis labels in wavelength units of μm shown along top panels for convenience.

Turning to the soluble absorption spectra, as mentioned in Section 3.3 there are broad spectral features present in *σ*_MeOH‐abs_ and *σ*_DI‐abs_ that neither parameterization captures. Figure 11 shows the residuals to the linear and second‐order polynomial fits for the four examples of *σ*_MeOH‐abs_ spectra in Figure 4 aside from the one in Figure 6. Here, as in Figure 6, the blue residual curves illustrate the improvement that second‐order fits provide in capturing spectral curvature. In addition, the second‐order fit residual is shown twice with the second version rescaled to highlight small spectral features that depart from the curvature of the full spectral fit. It is notable that these features differ between fires. Although the departures from a smooth second‐ order fit are subtle, they hint at the aforementioned superposition of contributions from different chromophores such that the curvature of any given spectrum arises from the sum of those contributions. A similar result is found from the residuals of the example *σ*_DI‐abs_ spectra (Figure S14 in Supporting Information S1). Further investigation is needed to evaluate the robustness of the result. It is likely that there are better mathematical approaches to resolving the various contributions across the spectrum than residuals to second‐order fits. However, these examples serve to illustrate the potential for deriving such information from in situ hyperspectral data.

### 4.3 Chromophoric Complexity, Atmospheric Evolution, Expanding Conception of BrC

The entwined contributions to BBOA composition that depend on fuels, fuel components, fuel water content, and combustion processes produce aerosols that possess different proportions of important chromophores. For example, sinapaldehyde and coniferaldehyde are inversely proportional in lignin pyrolysis products from angiosperms versus gymnosperms, because lignin itself is comprised of different monomers that occur in varying amounts in different plant species (Fleming et al., 2020). The proportion of anhydrosugars (e.g., levoglucosan, mannosan, and galactosan) from the pyrolysis of cellulose and hemicellulose differs between fuel types (e.g., softwoods, hardwoods, rice straw, peat) (e.g., Engling et al., 2014) and fuel components (e.g., stems, branches, needles) (e.g., Sullivan et al., 2008). However, Jayarathne et al. (2018) did not find consistent ratios of these anhydrosugars in peat burning emissions, and further they found that the emission profile from a plume from combined burning of peat and surface vegetation differed significantly from those generated from peat combustion alone. Such results suggest different fuels produce different BBOA composition, yet the laboratory study of Saleh et al. (2014) found burn conditions were a more important determinant in absorptivity than fuels. They noted, however, that these are not entirely separable as grasses tend to burn more completely than boreal forests that tend to smolder. Fuel water content is also likely to be an important determinant in the prevalence of the distillation products that contribute to BrC.

This complexity in chromophoric composition likely accounts for the inconsistent variability observed in 0.365 μm BrC evolution of aqueous extracts of BBOA in Lagrangian plume sampling from the Twin Otter during FIREX‐AQ (Washenfelder et al., 2022). To look at evolution of BrC absorption over time or to relate it to other observations of chemical, optical, or microphysical parameters it is convenient to select one wavelength from the BrC spectrum; 0.365 μm is the wavelength typically used for this purpose. Washenfelder et al. (2022) determined plume age both in terms of chemical and physical age, and observed BBOA evolution over the first 5 hr after emission (Washenfelder et al., 2022). O:C ratios varied between the flights, but oxidation of BBOA consistently increased with plume age for all of them. In contrast, the evolution with age of the mass absorption coefficient (MAC) at 0.365 μm varied from one flight to the next: most exhibited increasing or flat trends, while some exhibited modest decreases. Both observations (variable but increasing O:C and inconsistent trends in BrC) are consistent with a varying suite of chromophores for each plume where oxidation alters the chromophores differently across the set of plumes. Looking just at photolytic degradation of the filter samples (i.e., complete set of smoke aerosols from a given burn) from their laboratory study, Fleming et al. (2020) found that the absorption changes varied by fuel type, fuel components burned, and combustion condition, consistent with the variable trend observed by Washenfelder et al. (2022). Examining the photodegradation of individual chromophores, Fleming et al. (2020) found that lignin‐derived and distillation (specifically, flavonoids) products tended to have the longest lifetimes. This suggests atmospheric photooxidation will shift the proportion of chromophores with age in the plume.

BrC is typically thought of in terms of its contributions to absorption in the UV and short visible wavelength range such that it is routinely represented by absorptivity at 0.365 μm, but perhaps the concept of BrC should be broadened to include organic aerosol absorption at much longer wavelengths. Fleming et al. (2020) found the recalcitrant absorption in their irradiated filter samples extended to 0.7 μm, even though the absorption of the extractable fraction from those samples was below detection for *λ* >0.55 μm. In addition to the absorption spectrum of tarballs extending into the IR range, recent photoacoustic measurements have found excess absorption at 0.66 μm above that which can be attributed to BC (Adler et al., 2019; Mason et al., 2018). The excess absorption has been found to exceed that which can be attributable to lensing effects, hence, unidentified organic chromophores were responsible for the remainder (Adler et al., 2019; Mason et al., 2018). Indeed, Adler et al. (2019) discuss the possibility that tarballs may be responsible for the excess absorption at 0.66 μm, although they note further research is needed to quantify any contribution they may make. The observation here that *σ*_MeOH‐abs_ at 0.7 μm exceeded the limit of detection by 2–3 orders of magnitude supports the presence of organic chromophores at long visible wavelengths. This more expansive view and the fire‐to‐fire variability shown in this data set may be helpful in reframing how to think about biomass burning BrC.

These findings, indicating the presence of organic chromophores that absorb throughout the visible wavelength range, have important implications for calculations that partition measured absorption into fractions contributed by BC and BrC. Such calculations assume that all absorption observed at a long visible wavelength (e.g., 0.66 μm as in Zhang et al. (2017)) is due to BC alone (allowing for lensing effects from coatings). An Ångström exponent of 1 is then used to calculate the BC fraction over the rest of 0.3–0.7 μm spectral range. The difference between measured absorption and the calculated BC fraction is attributed to BrC. However, the presence of externally mixed organic aerosol chromophores contributing to the total measured absorption at long visible wavelengths, in addition to BC, suggest this method will overestimate the BC fraction, leading to underestimated BrC throughout the 0.3–0.7 μm spectral range.

### 4.4 New Opportunities Provided by Hyperspectral Observations

The vast number of organic compounds that comprise BBOA mass and the small, yet still numerous, subset of chromophores, each making small fractional contributions to the total absorptivity suggest that identifying all individual chromophores and determining their atmospheric fate will be a daunting task. However, the clustering of data points from the five fires in (*a*_1_, *a*_2_) maps (Figure 7) suggest that curvature captures different chromophores among the fires that drive spectral curvature. Leveraging the information in curvature may provide a new tool to better predict atmospheric evolution of MAC in biomass burning plumes from that provided by 0.365 μm alone.

Classifying individual chromophores that can be identified from soluble aerosol extracts into the four groups of Fleming et al. (2020) may offer a useful simplification to look for spectral fingerprints (whether spectral features or overall curvature) of relative contributions from dehydration and distillation, pyrolysis, flaming, and atmospheric processing. Perhaps different spectral fingerprints can distinguish boreal, temperate, and tropical ecosystem fires from forests, savannahs, grasslands, peat, and agricultural burns. Even though chromophores represent a small fraction of the BBOA mass their fingerprints will likely be useful for interpretation of sources and atmospheric processing of the BBOA mass in smoke plumes more broadly.

One way to approach this problem would be to develop lookup tables for models on the basis of spectral fingerprints for different types of BBOA based on ecosystem (biofuels), fire type (e.g., peat, surface, crown) and intensity (flaming/smoldering), and ambient conditions (meteorology, topography, water content/drought stress of plants) for which sufficient spectra have been acquired to parameterize typical spectral characteristics. Another would be to apply modern computational tools such as machine learning to better capture the complexity of measured spectra and their evolution downwind of sources. As we await sufficient accumulation of hyperspectral data to pursue more advanced representations of ambient atmospheric aerosol optical properties, second order polynomials can be used to reduce errors that arise from linear fits. Even so, we should strive to measure the wavelength range(s) of interest to avoid the need to extrapolate much beyond the measured range. Finally, sufficient spectral resolution is important to capture the spectral features evident in this data set.

That this is worth doing is due to the striking result here: that the spectra of the five fires separate in curvature space. To better understand the complexity of aerosol optical properties and how they evolve in the atmosphere requires moving beyond the Ångström exponent by leveraging the fine spectral resolution that can now be obtained by various hyperspectral measurement techniques to better link detailed optical information to underlying aerosol chemistry and microphysics. Adopting a more expansive view of BrC to include long visible wavelength chromophores will help resolve discrepancies between expected spectral characteristics from BC and short‐wavelength‐BrC formulations to what is found in current observations. More closely capturing real spectral characteristics in the near and far fields of smoke plumes is anticipated to improve our understanding of the modifications of the light field influencing photolysis rates in the UV to better characterization of aerosol composition and its evolution to improving representation of long visible into near IR absorption and its role in aerosol climate effects. Ultimately, improved understanding of in situ aerosol optical spectra is expected to lead to expanded capabilities to interpret and utilize hyperspectral data from on orbit sensors beyond what is possible from Ångström exponents.

## 5 Conclusions

Hyperspectral (0.3–0.7 μm) measurements of four sets of in situ atmospheric aerosol optical properties (*σ*_ext_, *σ*_abs_, *σ*_MeOH‐abs_, and *σ*_DI‐abs_) were obtained during the FIREX‐AQ field campaign, July–August 2019, from NASA Langley's ground‐based MACH‐2 mobile laboratory deployed across the western United States. Data from five fires are reported, including calculated hyperspectral *ω* from measured *σ*_ext_ and *σ*_abs_. Hyperspectral *ω* over such a wide wavelength range is rare in the literature. The results here reveal different spectral characteristics between the fires. Differences in biofuels, distances from the fire front, fire intensity metrics (ΔBC/ΔCO and ΔBC/ΔPM_2.5_), and composition based on FT‐IR functional groups among all five fires were discussed.

All the measured logarithmically transformed spectra were better fit by second‐order polynomials than traditional Ångström exponents. The observed spectral curvature indicates the spectral shape is not wavelength independent and, hence, measured values of these optical properties depend on the wavelength(s) of the measurements. This needs to be taken into consideration when comparing data measured at different wavelengths or across differing spectral ranges. Solvent effects on the measured *σ*_MeOH‐abs_ and *σ*_DI‐abs_ spectra suggest spectral *σ*_abs_ should be measured, when possible, to accurately represent ambient aerosol absorption spectra.

Taking advantage of the linear (*a*_1_) and second‐order (*a*_2_) coefficients of the polynomial fits, 2‐dimensional maps were created plotting the coefficients from each fit. This revealed the striking result that the coefficients of samples from a given fire tended to cluster together. That is, the spectral variability of aerosols from a given fire are similar to each other and somewhat distinct from the other fires. Further, separation was observed in this curvature space for samples that exhibited the same values of Ångström exponents. This suggests the second‐order parameterization contains more information than the single Ångström exponent parameter, specifically that spectral curvature in absorption is sensitive to underlying chromophores that differ across fires.

Recent research conducted in the laboratory and in the field suggests that the suite of chromophoric organic compounds that contribute to BrC is likely large and highly variable due to differences within the biomass fuels, across different fuel components (duffs, litter, stems, branches, and leaves), fuel water content, the heterogeneous combustion processes that decompose the fuel molecules into smaller MWt products, and the atmospheric processing that occurs after emission. These complexities are exacerbated by environmental conditions such as wind direction and speed (i.e., heading vs. backing fires), topography, and diurnal variations in temperature, relative humidity, and photooxidation pathways. Preliminary results here suggest spectral curvature and subtle deviations (i.e., spectral features observed in soluble aerosol extracts) from the mathematical fits better capture the optical variability driven by these diverse underlying properties and influences.

Ångström exponents and second‐order polynomial fits are both empirical representations of observations. They are not derived from first principles of underlying microphysical and chemical properties of the aerosols. Hence, other mathematical tools may prove to be more appropriate to fully exploit the spectral information contained in the type of data sets shown here. It is clear from these results that hyperspectral data offer new avenues for exploration that may enable better representations of the underlying drivers of spectral variability from BBAs across ecosystem types under different influences.

BBOA chromophores contribute across the full spectral range measured here, 0.3–0.7 μm. This has important implications for calculations that partition BC and BrC contributions to absorption spectra based on the assumption that all absorption at long visible wavelengths is due to BC, leading to an overestimate of the BC fraction and an underestimate of the BrC fraction. The spectral curvature is sensitive to BBOA chromophore contributions across the entire spectral range. Perhaps spectral curvature can be used to provide a more expansive characterization of BrC that includes both low MWt short‐lived chromophores driving absorption in the UV, as well as high MWt longer‐lived recalcitrant chromophores that absorb throughout the UV‐visible range. Incorporating routine hyperspectral in situ atmospheric aerosol measurements to fully capture spectral curvature and variability is expected to not only improve our scientific understanding of these aerosols, but by moving beyond Ångström exponents it will allow for more expansive interpretation of remote sensing data sets as well.

## Conflict of Interest

The authors declare no conflicts of interest relevant to this study.

## Supporting information

Supporting Information S1

## Data Availability

All data shown here are available from the Fire Influence on Regional to Global Environments and Air Quality (FIREX‐AQ) data archive: https://doi.org/10.5067/ASDC/FIREXAQ_SurfaceMobile_MACH2_InSitu_Data_1 (NASA/LARC/SD/ASDC, 2020). FIREXAQ_SurfaceMobile_MACH2_InSitu_Data are in situ measurements collected via the NASA Langley Aerosol Research Group mobile platform (MACH‐2) during FIREX‐AQ. See Section 2 for the specific measurements reported here.
