# Supplementary material for: Beyond the Ångström Exponent: Probing Additional Information in Spectral Curvature and Variability of In Situ Aerosol Hyperspectral (0.3–0.7 μm) Optical Properties
Source: J Geophys Res Atmos. 2022 Nov 3;127(21):e2022JD037201. doi: 10.1029/2022JD037201 (PMC9787633; doi:10.1029/2022JD037201)
Supplement: Supplementary file 1 — Supporting Information S1 [file JGRD-127-e2022JD037201-s001.docx]

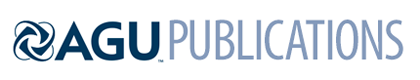


*Journal of Geophysical Research: Atmospheres*

Supporting Information for

Beyond the Ångström Exponent: Probing Additional Information in Spectral Curvature and Variability of in situ Aerosol Hyperspectral (0.3-0.7 µm) Optical Properties

Carolyn E. Jordan^1,2^, Bruce E. Anderson^2^, John D. Barrick^2,3^, Dani Blum^4^, Kathleen Brunke^5^, Jiajue Chai^4^, Gao Chen^2^, Ewan C. Crosbie^2,3^, Jack E. Dibb^6^, Ann M. Dillner^7^, Emily Gargulinski^1,2^, Charles H. Hudgins^2,3^, Emily Joyce^4^, Jackson Kaspari^6^, Robert F. Martin^2^, Richard H. Moore^2^, Rachel O’Brien^8^, Claire E. Robinson^2,3,8^, Gregory L. Schuster^2^, Taylor J. Shingler^2^, Michael A. Shook^2^, Amber J. Soja^1,2^, Kenneth L. Thornhill^2,3^, Andrew T. Weakley^7^, Elizabeth B. Wiggins^2^, Edward L. Winstead^2,3^, and Luke D. Ziemba^2^

^1^National Institute of Aerospace, Hampton, VA

^2^NASA Langley Research Center, Hampton, VA

^3^Science Systems and Applications Inc., Hampton, VA

^4^Brown University, Providence, RI

^5^Christopher Newport University, Hampton, VA

^6^University of New Hampshire, Durham, NH

^7^University of California – Davis, CA

^8^William & Mary, Williamsburg, VA

Corresponding author: Carolyn Jordan ([Carolyn.Jordan@nasa.gov)](mailto:Carolyn.Jordan@nasa.gov))

**Contents of this file**

Sections S1 to S9

Figures S1 to S14

Tables S1 to S3

**Introduction**

This supplement provides details on MACH-2 filter samples, methods, and data analysis in support of the main text as follows:

**Section S1.** MACH-2 description and sample information.

**Section S2.** Method to flag and exclude data due to generator exhaust contamination

**Section S3.** Estimation of background values of CO, BC, and PM_2.5_

**Section S4.** Additional details on filter sampling methods.

**Section S5.** Obtaining quantitative total absorption spectra from quartz filter punches

**Section S6.** Histograms of linear and second-order polynomial fit residuals for entire data set

**Section S7.** Spectral difference between aerosol- and solution-phase of nigrosine

**Section S8.** Partial spectra, curvature and axis of symmetry mapping in (*a_1_*,*a_2_*) coordinates

**Section S9.** Additional examples of spectral features evident in *σ_DI-abs_* spectra

**Section S1.** MACH-2 description and sample information.

Figure S1 provides a photo of MACH-2 with a brief description of the sampling inlet position with respect to the generator. Table S1 lists the filter samples presented in the main text, with sampling times and locations. This set excludes filter samples contaminated by the generator exhaust (see Section S2 for details on the contamination screening). Overall, 22% of the sampled filters were removed (Table S2), leaving 40 filters in the final data set (Table S1). The filter sampling time (Table S1) is provided in both UTC and local time (multiple time zones were visited, so there is not a single offset between them). Filter samples were collected while MACH-2 was parked, hence, the latitude, longitude, and altitude coordinates are fixed for each sample (Table S1).


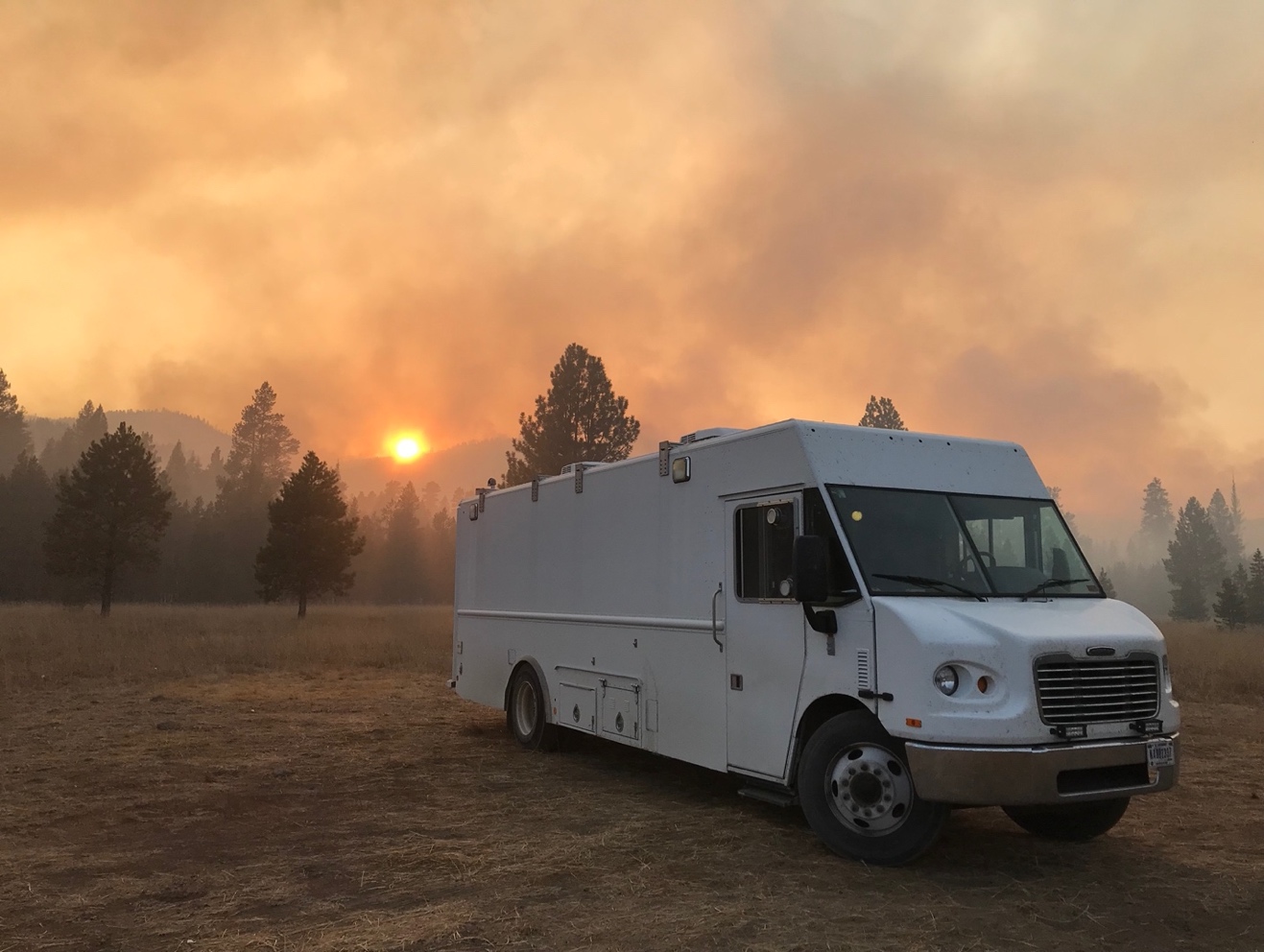


Figure S1. MACH-2 sampling at the 204 Cow fire in Oregon. Two HiVol filter samplers can be seen mounted to the passenger door window (white circles are quartz filters). The sampling inlet for all other measurements is just visible on top of the truck at the rear of the passenger side. The diesel generator used to run the equipment in the field is in a compartment on the driver’s side, opposite the two compartments visible in this photo. The SpEx instrument, along with the 47 mm filter samplers, sample off the same manifold inside the laboratory. Photo credit: B. E. Anderson.

Table S1. Filter numbers, fire names and number, filter sampling start and stop times in UTC and local time, and measurement location.

| Filter # | Fire  Name | Fire # | Date & Time (UTC) | | Date & Time (Local Time) | | Latitude (°N) | Longitude (°W) | Elevation (m) |
| --- | --- | --- | --- | --- | --- | --- | --- | --- | --- |
|  |  |  | Start | Stop | Start | Stop |  |  |  |
| 1 | Wms Flts | 3 | 8/4/19 4:55 | 8/4/19 8:35 | 8/3/19 21:55 | 8/4/19 1:35 | 48.10088 | 118.20834 | 578 |
| 2 | Wms Flts | 3 | 8/4/19 23:23 | 8/5/19 1:38 | 8/4/19 16:23 | 8/4/19 18:38 | 47.97105 | 118.29726 | 721 |
| 3 | Wms Flts | 3 | 8/5/19 1:53 | 8/5/19 3:53 | 8/4/19 18:53 | 8/4/19 20:53 | 47.98606 | 118.28885 | 738 |
| 4 | Wms Flts | 3 | 8/5/19 3:57 | 8/5/19 7:39 | 8/4/19 20:57 | 8/5/19 0:39 | 47.98606 | 118.28885 | 732 |
| 5 | Wms Flts | 3 | 8/5/19 7:53 | 8/5/19 13:20 | 8/5/19 0:53 | 8/5/19 6:20 | 47.98606 | 118.28885 | 727 |
| 6 | Wms Flts | 3 | 8/6/19 20:03 | 8/6/19 22:38 | 8/6/19 13:03 | 8/6/19 15:38 | 48.07076 | 118.22604 | 624 |
| 7 | Wms Flts | 3 | 8/7/19 4:10 | 8/7/19 10:12 | 8/6/19 21:10 | 8/7/19 3:12 | 47.96096 | 118.30758 | 509 |
| 8 | Wms Flts | 3 | 8/7/19 10:26 | 8/7/19 13:55 | 8/7/19 3:26 | 8/7/19 6:55 | 47.96096 | 118.30758 | 498 |
| 9 | Nethker | 4 | 8/11/19 3:45 | 8/11/19 4:46 | 8/10/19 21:45 | 8/10/19 22:46 | 45.25664 | 115.89639 | 1865 |
| 10 | Nethker | 4 | 8/11/19 4:56 | 8/11/19 6:16 | 8/10/19 22:56 | 8/11/19 0:16 | 45.25664 | 115.89639 | 1862 |
| 11 | Nethker | 4 | 8/11/19 6:22 | 8/11/19 7:32 | 8/11/19 0:22 | 8/11/19 1:32 | 45.25664 | 115.89639 | 1860 |
| 12 | Nethker | 4 | 8/11/19 11:46 | 8/11/19 13:29 | 8/11/19 5:46 | 8/11/19 7:29 | 45.25600 | 115.89639 | 1889 |
| 13 | Nethker | 4 | 8/12/19 3:49 | 8/12/19 5:42 | 8/11/19 21:49 | 8/11/19 23:42 | 45.25664 | 115.89639 | 1821 |
| 14 | Nethker | 4 | 8/12/19 5:46 | 8/12/19 11:30 | 8/11/19 23:46 | 8/12/19 5:30 | 45.25664 | 115.89639 | 1819 |
| 15 | Nethker | 4 | 8/12/19 11:36 | 8/12/19 12:40 | 8/12/19 5:36 | 8/12/19 6:40 | 45.25664 | 115.89639 | 1814 |
| 16 | Nethker | 4 | 8/12/19 12:44 | 8/12/19 13:54 | 8/12/19 6:44 | 8/12/19 7:54 | 45.25664 | 115.89639 | 1811 |
| 17 | Nethker | 4 | 8/14/19 3:44 | 8/14/19 4:46 | 8/13/19 21:44 | 8/13/19 22:46 | 45.25663 | 115.89639 | 1816 |
| 18 | Nethker | 4 | 8/14/19 4:50 | 8/14/19 6:00 | 8/13/19 22:50 | 8/14/19 0:00 | 45.25665 | 115.89639 | 1812 |
| 19 | Nethker | 4 | 8/14/19 6:04 | 8/14/19 7:11 | 8/14/19 0:04 | 8/14/19 1:11 | 45.25664 | 115.89639 | 1810 |
| 20 | Nethker | 4 | 8/16/19 4:06 | 8/16/19 5:09 | 8/15/19 22:06 | 8/15/19 23:09 | 45.25750 | 115.89323 | 1843 |
| 21 | Nethker | 4 | 8/16/19 5:12 | 8/16/19 6:12 | 8/15/19 23:12 | 8/16/19 0:12 | 45.25775 | 115.89194 | 1835 |
| 22 | Nethker | 4 | 8/16/19 6:15 | 8/16/19 10:53 | 8/16/19 0:15 | 8/16/19 4:53 | 45.25780 | 115.89162 | 1838 |
| 23 | Nethker | 4 | 8/16/19 10:57 | 8/16/19 12:13 | 8/16/19 4:57 | 8/16/19 6:13 | 45.25781 | 115.89161 | 1843 |
| 24 | Ltl. Bear | 5 | 8/21/19 1:01 | 8/21/19 2:26 | 8/20/19 19:01 | 8/20/19 20:26 | 37.60989 | 112.26301 | 2411 |
| 25 | Ltl. Bear | 5 | 8/21/19 12:20 | 8/21/19 13:22 | 8/21/19 6:20 | 8/21/19 7:22 | 37.60939 | 112.25439 | 2360 |
| 26 | Ltl. Bear | 5 | 8/21/19 13:25 | 8/21/19 14:50 | 8/21/19 7:25 | 8/21/19 8:50 | 37.60939 | 112.25439 | 2360 |
| 27 | Castle | 6 | 8/23/19 3:51 | 8/23/19 4:52 | 8/22/19 20:51 | 8/22/19 21:52 | 36.56716 | 112.17277 | 2674 |
| 28 | Castle | 6 | 8/23/19 4:55 | 8/23/19 5:47 | 8/22/19 21:55 | 8/22/19 22:47 | 36.56717 | 112.17277 | 2673 |
| 29 | Castle | 6 | 8/23/19 5:51 | 8/23/19 6:38 | 8/22/19 22:51 | 8/22/19 23:38 | 36.56716 | 112.17277 | 2670 |
| 30 | Castle | 6 | 8/23/19 6:41 | 8/23/19 7:49 | 8/22/19 23:41 | 8/23/19 0:49 | 36.57891 | 112.17482 | 2661 |
| 31 | 204 Cow | 7 | 8/26/19 21:28 | 8/26/19 22:34 | 8/26/19 14:28 | 8/26/19 15:34 | 44.24799 | 118.39780 | 1701 |
| 32 | 204 Cow | 7 | 8/26/19 22:37 | 8/26/19 23:43 | 8/26/19 15:37 | 8/26/19 16:43 | 44.24460 | 118.39403 | 1708 |
| 33 | 204 Cow | 7 | 8/26/19 23:46 | 8/27/19 0:47 | 8/26/19 16:46 | 8/26/19 17:47 | 44.24458 | 118.39402 | 1713 |
| 34 | 204 Cow | 7 | 8/28/19 1:04 | 8/28/19 1:58 | 8/27/19 18:04 | 8/27/19 18:58 | 44.29041 | 118.40681 | 1798 |
| 35 | 204 Cow | 7 | 8/28/19 2:01 | 8/28/19 2:49 | 8/27/19 19:01 | 8/27/19 19:49 | 44.29042 | 118.40682 | 1803 |
| 36 | 204 Cow | 7 | 8/28/19 2:52 | 8/28/19 3:49 | 8/27/19 19:52 | 8/27/19 20:49 | 44.29041 | 118.40681 | 1837 |
| 37 | 204 Cow | 7 | 8/28/19 3:52 | 8/28/19 4:40 | 8/27/19 20:52 | 8/27/19 21:40 | 44.29041 | 118.40681 | 1902 |
| 38 | 204 Cow | 7 | 8/28/19 4:44 | 8/28/19 5:33 | 8/27/19 21:44 | 8/27/19 22:33 | 44.29042 | 118.40681 | 1948 |
| 39 | 204 Cow | 7 | 8/28/19 5:36 | 8/28/19 6:27 | 8/27/19 22:36 | 8/27/19 23:27 | 44.29041 | 118.40681 | 1979 |
| 40 | 204 Cow | 7 | 8/28/19 6:30 | 8/28/19 7:34 | 8/27/19 23:30 | 8/28/19 0:34 | 44.29041 | 118.40681 | 2006 |

**Section S2.** Method to flag and exclude data due to generator exhaust contamination

Filter samples were collected while parked (to limit contamination from other vehicles on roadways) with MACH-2 oriented to avoid sampling its own generator exhaust. However, shifting winds sometimes led to incidents of sampling that exhaust. These were identified and flagged by adapting the methodology of Halliday et al. (2019). In short, a paired test was performed comparing the maximum vs. minimum values over a 61 s interval spanning a 1 s data point using NO_2_ (delta > 20 ppbv; NO_2_ analyzer, Los Gatos Research, San Jose, CA) and particle number concentration (CN, delta > 45,000 particles cm^-3^; Condensation Particle Counter model 3775, TSI Inc., Shoreview, MN). If both delta thresholds were exceeded (i.e., a large spike in both values over the 61 s interval), then that 1 s data point was flagged as contaminated (generator exhaust flag, GEF = 1). During periods when NO_2_ data was missing (either the instrument was zeroing or was offline), a secondary paired test was performed using CO_2_ (delta > 80 ppmv; LI-840 CO_2_/H_2_O gas analyzer, LI-COR Biosciences, Lincoln, NE) with CN instead. In performing the secondary test, if either CO_2_ or CN is missing, GEF is set to NaN. Filter samples were collected over ~1-6 hours. Using 1 s nephelometer (IN101, AirPhoton, Baltimore, MD) and Tricolor Absorption Photometer (TAP, model 2901, Brechtel, Hayward, CA) data at 0.53 µm, the mean single scattering albedo (*ω*) was calculated using all the data from a filter sampling interval versus that calculated excluding flagged data. In addition, the mean GEF was calculated for each filter. Figure S2 shows the plot of the mean *ω* for each filter calculated using either all values or only GEF=0 (i.e., no exhaust contamination) values. These plots are colored by the mean GEF for each filter.

Figure S2. Comparison of mean *ω* (0.53 µm) calculated over each filter sampling interval using all 1 s data versus the mean calculated using only data points when GEF=0. Filters with values that fall off the 1:1 line (left panel, all filter data points) are excluded from the final data set (right panel).

Nominally as the fraction of GEF=1 increases (i.e., mean GEF ≥ 0.1), the filter mean *ω* falls further off the 1:1 line with the GEF=0 *ω*. But this is not strictly true. There are two cases where a fraction > 0.2 appears on the 1:1 line. In those cases, the TAP data was missing for about one third of the sampling period and it was during that time when the exhaust interceptions were most prevalent, so the mean absorption (and hence, *ω*) did not reflect the complete sampling period. Conversely, there were a few points with < 0.2 of the data flagged that fell well off the 1:1 line. In these cases, rather than a series of fleeting interceptions over the sampling interval, more intense interceptions persisted long enough to skew the mean absorption such that a large difference was found from the *ω* calculated from the GEF=0 data. Only the filters shown in the right panel have been retained for further analyses of wildfire smoke properties. The percentage of filters excluded due to generator exhaust contamination are listed in Table S2.

Table S2. Percentage of filters excluded from fire analyses due to contamination by MACH-2 generator exhaust.

| *Fires* | *# of Filters Collected* | *# Contaminated* | *% Excluded* |
| --- | --- | --- | --- |
| Shady | 2 | 2 | 100 |
| Williams Flats | 8 | -- | 0 |
| Nethker | 20 | 5 | 25 |
| Little Bear | 5 | 2 | 40 |
| Castle | 4 | -- | 0 |
| 204 Cow | 12 | 2 | 17 |
| *Total* | *51* | *11* | *22* |

**Section S3.** Estimation of background values of CO, BC, and PM_2.5_

Filter means and background values of CO, BC and PM_2.5_ are provided in Table S3. Although filter samples were only collected when MACH-2 was parked, most of the instruments were running as MACH-2 was driven to and from smoke plumes. The time series of CO, BC, and PM_2.5_ for each fire was plotted and a horizontal line was used to approximate a typical background value outside of the smoke plumes (e.g., Fig. S3).

Table S3. Filter means (± 1 standard deviation) from 1 Hz measurements of gas-phase volume mixing ratios of CO (ppmv), along with aerosol mass concentrations for BC and PM_2.5_ (µg m^-3^). Background (Bkg) values estimated for each fire are also provided. Gray font used to highlight the only filter sample (FN-5) collected in predominantly background conditions.

| Filter  # | CO ± 1 s.d. (ppmv) | Bkg CO (ppmv) | BC ± 1 s.d. (µg m-3) | Bkg BC (µg m-3) | PM_2.5_ ± 1 s.d. (µg m-3) | Bkg PM_2.5_ (µg m-3) |
| --- | --- | --- | --- | --- | --- | --- |
| 1 | 0.56 ± 0.12 | 0.2 | 4.01 ± 1.26 | 0.25 | 101.3 ± 33.0 | 10 |
| 2 | 0.48 ± 0.24 | 0.2 | 3.86 ± 1.50 | 0.25 | 81.8 ± 47.7 | 10 |
| 3 | 0.53 ± 0.01 | 0.2 | 3.73 ± 0.23 | 0.25 | 96.0 ± 6.2 | 10 |
| 4 | 0.38 ± 0.08 | 0.2 | 2.27 ± 0.75 | 0.25 | 57.6 ± 19.2 | 10 |
| 5 | 0.18 ± 0.02 | 0.2 | 0.21 ± 0.19 | 0.25 | 7.4 ± 4.8 | 10 |
| 6 |  | 0.2 | 3.05 ± 1.40 | 0.25 | 80.8 ± 37.8 | 10 |
| 7 | 0.36 ± 0.05 | 0.2 | 2.22 ± 0.56 | 0.25 | 48.9 ± 11.1 | 10 |
| 8 | 0.33 ± 0.03 | 0.2 | 1.52 ± 0.18 | 0.25 | 33.1 ± 3.3 | 10 |
| 9 | 12.40 ± 4.50 | 0.3 | 4.43 ± 1.04 | 0.5 | 574.9 ± 175.4 | 10 |
| 10 | 18.06 ± 4.65 | 0.3 | 7.50 ± 2.93 | 0.5 | 810.5 ± 177.8 | 10 |
| 11 | 14.58 ± 4.66 | 0.3 | 7.55 ± 1.51 | 0.5 | 719.8 ± 173.0 | 10 |
| 12 | 10.49 ± 2.02 | 0.3 | 6.04 ± 1.07 | 0.5 | 467.5 ± 81.0 | 10 |
| 13 | 5.89 ± 2.76 | 0.3 | 4.50 ± 1.56 | 0.5 | 321.8 ± 104.3 | 10 |
| 14 | 2.86 ± 1.81 | 0.3 | 1.96 ± 1.31 | 0.5 | 221.0 ± 99.5 | 10 |
| 15 | 12.41 ± 3.94 | 0.3 | 4.45 ± 0.75 | 0.5 | 658.2 ± 120.5 | 10 |
| 16 | 18.44 ± 5.51 | 0.3 | 5.31 ± 1.02 | 0.5 | 868.9 ± 186.8 | 10 |
| 17 | 5.54 ± 2.86 | 0.3 | 3.72 ± 1.31 | 0.5 | 314.1 ± 92.8 | 10 |
| 18 | 6.77 ± 2.91 | 0.3 | 4.13 ± 1.09 | 0.5 | 403.1 ± 110.1 | 10 |
| 19 | 8.63 ± 3.32 | 0.3 | 5.12 ± 1.29 | 0.5 | 516.6 ± 160.7 | 10 |
| 20 | 9.00 ± 6.88 | 0.3 | 1.44 ± 0.21 | 0.5 | 228.1 ± 145.2 | 10 |
| 21 | 12.52 ± 8.58 | 0.3 | 2.98 ± 1.18 | 0.5 | 386.7 ± 180.2 | 10 |
| 22 | 10.99 ± 2.98 | 0.3 | 2.81 ± 0.85 | 0.5 | 407.0 ± 109.5 | 10 |
| 23 | 12.33 ± 2.51 | 0.3 | 3.80 ± 0.93 | 0.5 | 502.1 ± 86.9 | 10 |
| 24 | 0.44 ± 0.23 | 0.2 | 3.27 ± 2.09 | 0.5 | 55.3 ± 32.3 | 10 |
| 25 | 5.58 ± 1.05 | 0.2 | 9.02 ± 0.70 | 0.5 | 355.0 ± 51.1 | 10 |
| 26 | 1.97 ± 1.71 | 0.2 | 3.05 ± 3.13 | 0.5 | 141.2 ± 109.6 | 10 |
| 27 | 2.17 ± 0.21 | 0.2 | 10.99 ± 0.49 | 0.5 | 299.8 ± 23.0 | 10 |
| 28 | 3.89 ± 0.98 | 0.2 | 12.64 ± 1.48 | 0.5 | 410.7 ± 60.6 | 10 |
| 29 | 3.82 ± 0.79 | 0.2 | 11.79 ± 0.55 | 0.5 | 402.6 ± 38.6 | 10 |
| 30 | 1.94 ± 0.76 | 0.2 | 9.21 ± 1.24 | 0.5 | 282.2 ± 93.0 | 10 |
| 31 | 4.43 ± 0.87 | 0.4 | 14.31 ± 1.03 | 1.0 | 441.1 ± 66.8 | 20 |
| 32 | 5.80 ± 1.30 | 0.4 | 15.27 ± 0.61 | 1.0 | 527.7 ± 38.3 | 20 |
| 33 | 6.61 ± 0.49 | 0.4 | 15.96 ± 0.79 | 1.0 | 518.3 ± 11.6 | 20 |
| 34 |  | 0.4 | 11.11 ± 4.85 | 1.0 | 520.1 ± 335.1 | 20 |
| 35 |  | 0.4 | 19.47 ± 1.25 | 1.0 | 1225.7 ± 153.1 | 20 |
| 36 |  | 0.4 | 18.96 ± 0.70 | 1.0 | 1242.2 ± 68.2 | 20 |
| 37 |  | 0.4 | 16.03 ± 1.34 | 1.0 | 897.3 ± 131.5 | 20 |
| 38 |  | 0.4 | 14.29 ± 1.17 | 1.0 | 800.0 ± 144.8 | 20 |
| 39 |  | 0.4 | 12.58 ± 0.88 | 1.0 | 565.4 ± 102.2 | 20 |
| 40 |  | 0.4 | 13.14 ± 0.36 | 1.0 | 620.6 ± 62.0 | 20 |

Figure S3. Example of graphical estimate of background values for CO (top), BC (bottom, left axis), and PM_2.5_ (bottom, right axis) for the Castle filter samples.

Figure S3 illustrates why this graphical approach was adopted in place of calculating 5^th^, 10^th^ or other fixed percentiles. The background value varied between fires due to varying ambient conditions. In the excess mixing ratio or aerosol concentration calculations for smoke plumes, they should represent the ambient non-plume background not the minimum values observed. For the Castle fire, in particular (Fig. S3), MACH-2 drove extensively around the area looking for a good location to sample the plume. There was a brief plume encounter between ~2:30 and 3:00 p.m. UTC on 8/22/2019, but none again until shortly before collection of FN-27 (Fig. S3). The rest of the day represented the local background which was somewhat variable with values in excess of the 5^th^ percentile. The lines shown were chosen to be somewhat greater than minimum values yet less than non-plume maxima (excluding brief spikes).

**Section S4.** Additional details on filter sampling methods.

All filters post-sampling were individually stored in their pre-sampling containers, wrapped in foil, sealed in Ziploc bags, and frozen until they could be analyzed in the laboratory post-mission.

*Teflon filters.*  FT-IR spectra were acquired in transmission mode over the range 4000 cm^-1^ - 420 cm^-1^ with a resolution of 4 cm^-1^. A total of 512 scans per sample were collected and averaged to improve the signal to noise ratio. The instrument optics and sample chamber were purged with CO_2_- and water-free air (PureGas LLC, Broomfield, CO) to minimize their contribution to the spectra. The resulting signal is converted to an absorption spectrum using the most recent empty chamber spectrum, acquired hourly. For more information on FT-IR analysis see Debus et al. (2019).

The methodology for the subsequent LWCC analyses of the Teflon filters required some modifications from Jordan et al. (2021b) due to the pandemic. First, the analyses were performed at UNH using a 250 cm optical path LWCC-3250 (World Precision Instruments, Sarasota, FL) instead of the 100 cm optical path instrument (LWCC-3100) at LaRC. Second, the 15 mL polypropylene centrifuge tubes (Corning 430052) used for the DI extractions were unavailable. Instead, 15 mL glass vials with Teflon caps (the same type used for the MeOH extractions) provided the best alternative among the available options. Blank tests following an extensive series of rinsing and soaking in DI provided acceptable blanks and lower limits of detection (LLOD = mean blank + 3*st. dev. of the mean). Note, there are no *σ_DI-abs_* spectra for the Williams Flats fire due to problems with the first alternate attempted in place of the centrifuge tubes.

A 10 mL extraction volume was used as before, but a series of dilutions was usually required for this filter set due to the high concentration of smoke aerosols sampled over 1-6 h. Sample spectra well above the LLOD were smoothly varying with little noise in the measurement, as was the case for all *σ_MeOH-abs_* spectra. However, for spectra that approached the LLOD noise was much more evident and led to intermittence in above detection data when blank corrected. This only occurred for the *σ_DI-abs_* set. To resolve this problem, the *σ_DI-abs_* spectra were smoothed via 0.01 µm boxcar smoothing prior to blank correction and the application of the LLOD test. Smoothing was not necessary for *σ_MeOH-abs_*(*λ*) prior to blank correction.

*Quartz filters.* The primary concern with this measurement for the FIREX-AQ filters was that they might be too overloaded to make a quantitative measurement. As discussed in Ogren et al. (2017), once the transmission of light through the filter drops to ~70% of that through a clean new filter the response of the measurement of aerosol absorption is no longer linear (see Section S5 for details). However, the wavelength dependence of the absorbance spectrum was found to not be a function of filter loading (Section S5). Even heavily loaded filters preserve the wavelength dependence of the spectrum observed for light loading. This feature of the measurement makes it possible to scale *σ_abs_*(*λ*) to the quantitative measurements provided by the TAP (model 2901) to obtain quantitative *σ_abs_* spectra. Since scaling was used to obtain the quantitative coefficients, no blank corrections were performed.

**Section S5.** Obtaining quantitative total absorption spectra from quartz filter punches


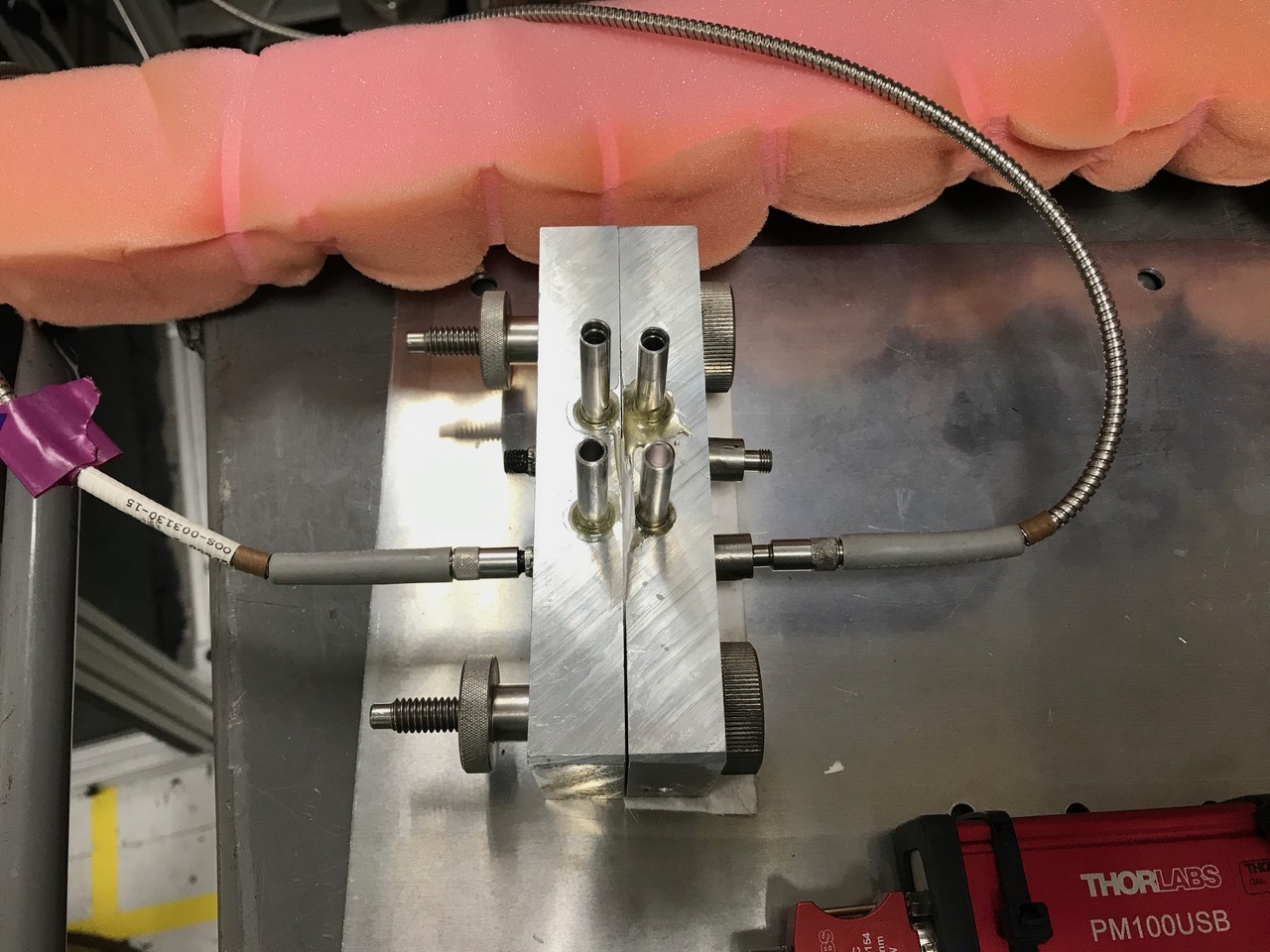


Figure S4. Filter holder designed for 47 mm quartz filters: one channel for reference, other for sample measurement for online operation. For this post-campaign study, a paper holder suspended 1 cm punches of sample filters in the beam of light using only one optic port.


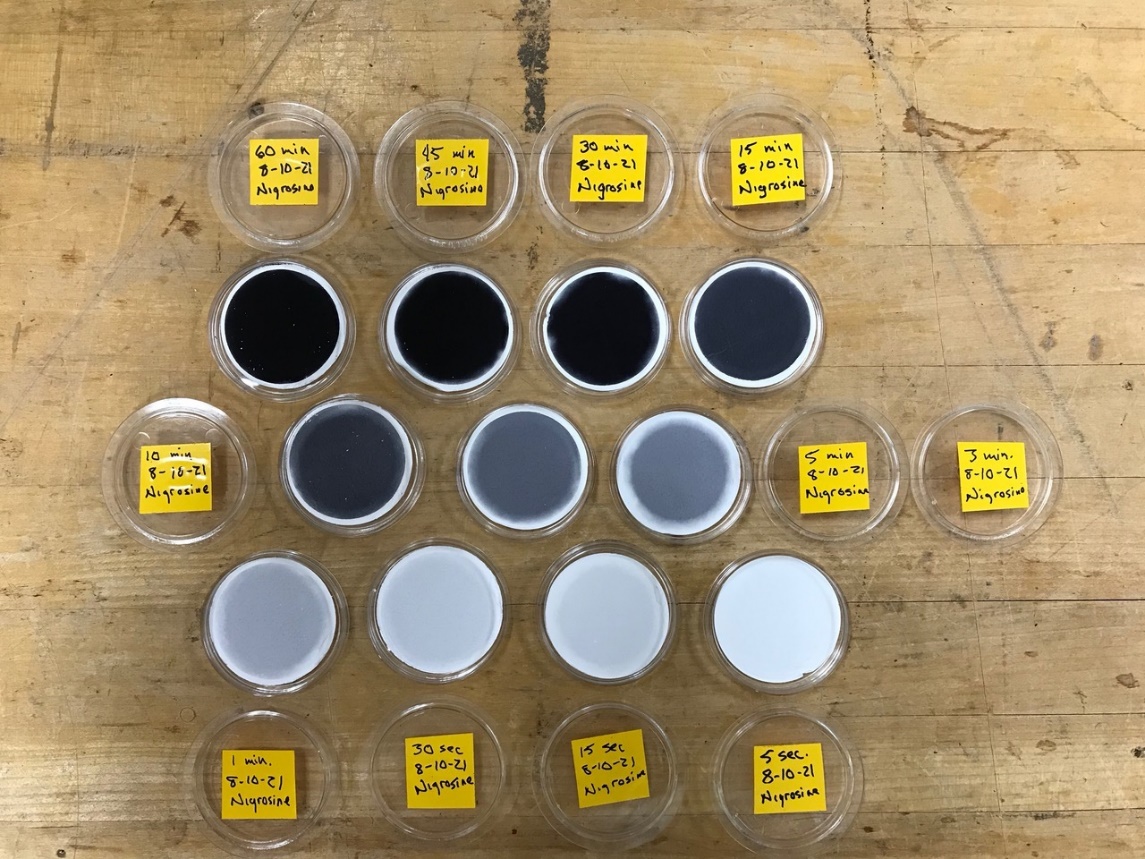


Figure S5. Series of filters loaded with nigrosine nebulized from a concentrated DI solution. Filters shown in petri dishes with labeled dish lids adjacent to indicate the elapsed sampling time for each filter. For example, filters loaded for 60, 45, 30, and 15 min (top, left to right) and for 1 min, 30 s, 15 s, and 5 s (bottom left to right).

As noted in Section S4, it was not certain whether it would be possible to obtain a quantitative measurement from the heavily loaded quartz filter punches from the campaign. To test this a series of filters was prepared in the laboratory using nigrosine dissolved in DI nebulized and sampled using extra filters from the same set of pre-baked quartz filters used during the field campaign (Fig. S5). A range of sampling times were used from 5 s to 60 min to test the linearity of the response in attenuation (*ATN*). The *ATN* of light through the filter is calculated from the ratio between the sample light intensity (*I_s_*) and the reference light intensity (*I_r_*) via Eq. S1 (Ogren et al., 2017),

$ATN= -LN\left( \frac{I_{s}}{I_{r}} \right)$ (S1)

Note, the dark spectrum was subtracted from the measured *I_s_* and *I_r_* prior to calculating *ATN*. Further, the mean from the pre- and post-sample *I_r_* (filter blank) was calculated and used in Eq. S1 to account for any drift in the lamp intensity between scans.

The measured intensities are shown in Fig. S6. Each test filter was measured 4 times with blanks measured in between starting with the least loaded filter (5 s) increasing through the entire set shown in Fig. S5 to the most heavily loaded filter (60 min). The measured spectra from each filter were highly reproducible from one measurement to the next. The blank spectra (high intensity black curves in Fig. S6) were also highly reproducible from one measurement to the next until the last two filters in the series (45 and 60 min) which were so heavily loaded that nigrosine smeared onto the aluminum block holder and from there onto the blank filter making it increasingly dark. It was not worth repeating the test for the last two as the results shown in Fig. S6 were sufficient to demonstrate a reproducible measurement and to check for linearity (Fig. S7).

Figure S6. All intensity spectra for the nigrosine on quartz filter tests. Four measurement repetitions for each filter show good reproducibility for each filter. See text for explanation of overlap of 5 s spectra set with atypically low reference (blank) spectra.

Figure S7. Select wavelengths for each *ATN* spectrum vs. accumulation time: a) all test filters, b) only filters with accumulation times of 15 min or less. Upper *ATN* limit of 0.3 indicated by the black horizontal line (b). Region for which there is a linear response limited by 0.3 limit (compare black angled line to guide the eye, hand drawn, to four shortest collection times, i.e., ≤ 1 min, to the horizontal line).


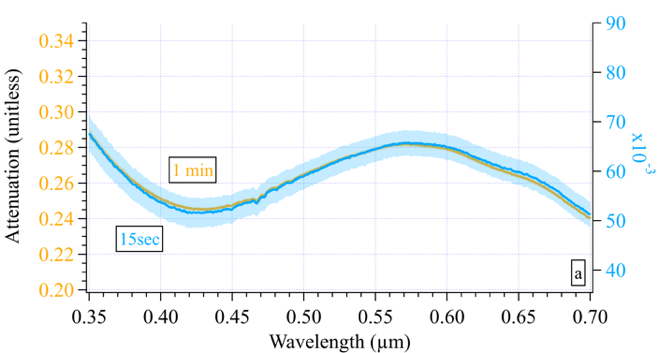

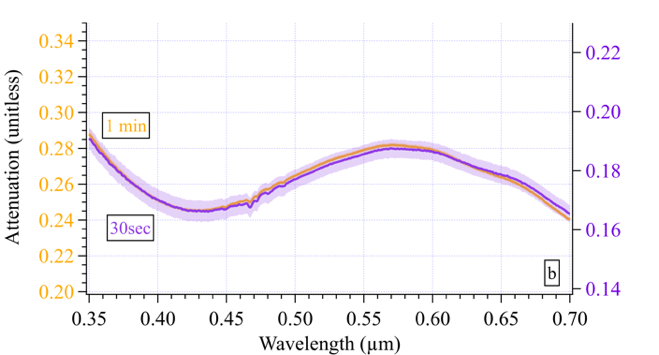


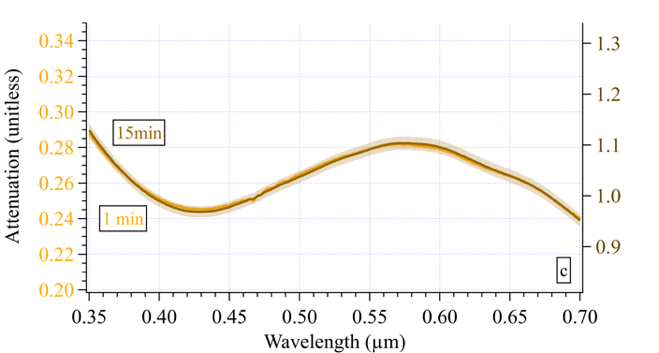

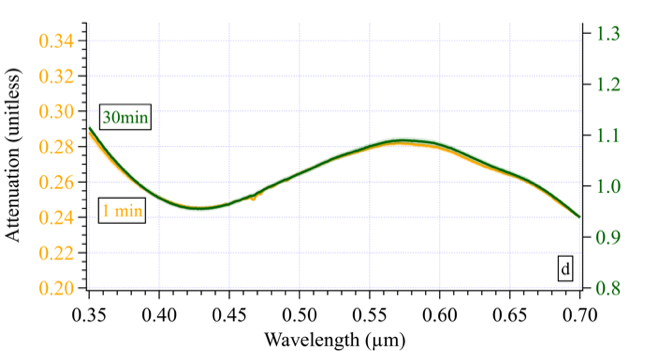


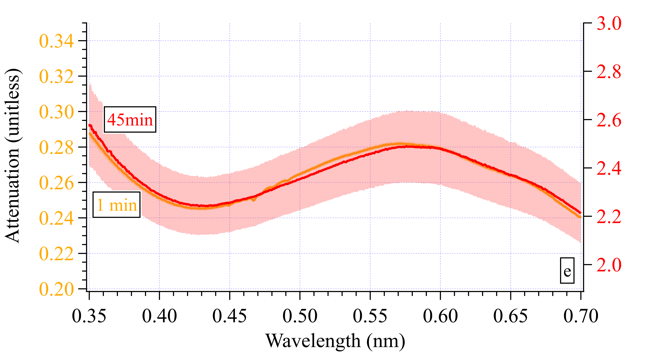

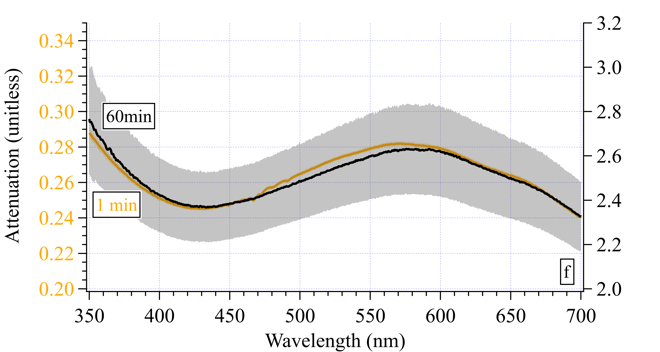


Figure S8. Mean (± 1 st. dev. shading) *ATN* (Eq. S1) spectrum from 1 min sample (gold curve, left axes all panels) with one of 6 other samples, right axes: a) 15 s, b) 30 s, c) 15 min, d) 30 min), e) 45 min, and f) 60 min to illustrate preservation of the wavelength dependence: with appropriate scaling the spectra match.

Using selected wavelengths, *ATN* versus accumulation time (Fig. S7) was used to test the linearity of the response to filter loading. Figure S7a shows all of the tests, including the 45 and 60 min loading samples. There are two possible interpretations of this figure. First, a smooth curve should fit all the data and something went wrong with the 30 min sample. Second, after an initial series exhibiting a linear response (up to ~1 min, Fig. S7b) increased loading was no longer linear with *ATN* approaching an asymptotic limit (~15-30 min for this series), where the extreme loading of the 45 and 60 min tests led to extreme values of *ATN*. The extremely low intensities for the 45 and 60 min tests (Fig. S6) suggests the second interpretation is more likely to be correct. In addition, an asymptotic limit is consistent with a similar asymptotic limit observed in absorbance spectra of highly absorbing liquids in liquid waveguide measurements. Future tests are planned to resolve this question but they await modifications to the optical path of the filter holder as will be explained below.

Figure S7b shows the 0.3 upper limit (equivalent to the lower limit of transmittance of 0.7 for PSAP and TAP instruments) is the appropriate limit for a linear response to aerosol loading on the filter here as well. All the FIREX-AQ 1 cm quartz

punches exceeded this limit for some portion of their *ATN* spectrum, which suggests a quantitative measurement is not possible. However, the *ATN* spectra from the nigrosine tests exhibited the same spectral shape (Fig. S8) in all cases. Note, that up to the 30 min loading test the standard deviations of the mean *ATN* spectrum (indicated by shading around the mean, Fig. S8) calculated for the four repeat measurements shown in Fig. S6 is small and with scaling the mean *ATN* spectrum for all test intervals match the 1 min mean spectrum. Surprisingly, even the extreme loading tests preserve the shape of the spectrum, albeit with minor deviations and much larger standard deviations of the means (Fig. S8).

Figure S9 illustrates the form of the intensity spectra measured for all the MACH-2 FIREX-AQ samples. Extremely low intensity in the samples is only observed near the 0.35 µm limit we applied to the measurement. This limit was unexpected compared to the typical intensity spectrum observed with the SpEx instrument (see Fig. S5 in Jordan et al. (2021a)). The low light intensity observed in the UV portion of the spectra of blank filters in the nigrosine test (Fig. S6) led us to replace the lamp. That resulted in a shift in the measured blank spectra, reducing the UV intensity further. Further tests revealed other problems in the optical path that needed to be addressed to resolve the problem. This work is underway and until it is complete, a further assessment of the results of Fig. S7a cannot be performed. Nonetheless, the preservation of the spectral shape across the filter loading tests (Fig. S8) and sample intensity spectra in the UV (Fig. S9) well represented by the 5-10 minute loading tests (Fig. S6) provides confidence that quantitative spectra may be obtained from overloaded filters if they can be scaled to a simultaneous quantitative absorption measurement. Here, the TAP measurements at 0.467, 0.528, and 0.652 µm, were used for this scaling. Note, the block was cleaned periodically throughout the sample measurements to prevent contamination of the blanks as in the 45 and 60 min nigrosine tests.

Figure S9. Intensity spectra measured for 204 Cow samples represent the observed spectral form for all samples. As with nigrosine, a series of four measurements were made per filter, alternating blank and sample spectra.

First, the attenuation coefficient (*σ_atn_*, m^-1^) is calculated from *ATN* (Eq. S1) for each filter mean via

$\sigma_{atn}=\frac{A}{Q}ATN$ (S2)

where, *Q* (m^3^) is the sample flow volume through the filter and *A* (m^2^) is the area of the filter (Ogren et al., 2017). Ordinarily, the absorption coefficient (*σ_ap_*, m^-1^) would then be calculated from Eq. S2 by correcting for changes in *ATN* caused by light scattering from

the particles on the filter, the multiple scattering and absorption of light within the filter medium, and the reduction of the multiple-scattering effect as the filter attenuation increases,

$\sigma_{ap}=0.85\frac{f\left( \tau\right)\sigma_{atn}}{K_{2}}-\frac{K_{1}\sigma_{sp}}{K_{2}}$ (S3)

Here, *K_1_* and *K_2_* are constants (=0.02±0.02 and 1.22±0.20, resp., Bond et al. (1999); Ogren (2010)), *σ_sp_* is the aerosol light-scattering coefficient adjusted to the wavelength of the absorption measurement, and *f(τ)* is the transmittance correction term, *f(τ)* = (1.0796*τ*+0.71)^-1^, where, *τ* is the normalized filter transmittance at time *t* relative to transmittance at the start of sampling (*t*=0), *τ* = (*I_s_*(*t*)/*I_r_*(*t*)) / (*I_s_*(0)/*I_r_*(0)) (Ogren et al., 2017). The time factor in *f(τ)* is required by the online nature of the TAP/PSAP instruments unlike the post-sampling filter measurements reported here. Since Eq. S3 cannot be used due to the high loading of the filters, the TAP data was paired to the 3 commensurate wavelengths in the filter measurements to scale the spectra. Note, the TAP data themselves were corrected with scattering from the AirPhoton nephelometer.

It is important to note that the correction terms in Eq. S3 are required to provide a quantitative measurement of absorption (Bond et al., 1999; Virkkula et al., 2005; Ogren, 2010; Virkkula, 2010; Muller et al., 2014; Stramski et al., 2015; Ogren et al., 2017; IOCCG, 2018). Corrections for filter absorption measurements differ across instruments due to measurement geometry (e.g., Stramski et al., 2015) and across targeted particle populations due to differing size distributions and absorption characteristics (e.g., Grenfell et al., 2011; Stramski et al., 2015; IOCCG, 2018). Hence, there are a couple of important caveats to the scaling approach used here: 1) the applicability of corrected visible data to correct the full spectral range of this data set (i.e., wavelength-dependence of corrections), and 2) the uncertainty introduced by scattering aerosols to the corrections in Eq. S3 (*ω*-dependence).

*Wavelength-dependence*. There is limited data available on UV absorption of atmospheric aerosols measured using this type of filter methodology, however, a lot of work has been done in this area by the ocean color community (e.g., Stramski et al. (2015) and references therein). They note that the correction scheme they derived for aquatic particles only applied to the visible portion of their hyperspectral measurement range (which spanned 0.30-0.85 µm). This was because comparisons between their filtered samples and the reference suspensions (in 1 cm cuvettes) that showed good agreement for all particle types in the visible range, did not extend to the UV. The UV portion of the absorption spectra of suspended minerals was well behaved and could be represented, but that of various living and detrital particles could not. They recommended dedicated studies to investigate how to calibrate and correct filter methods into the UV.

Similarly, it remains to be studied whether the correction scheme shown in Eq. S3 applies to the UV range of atmospheric particles, especially in the presence of brown carbon. Wavelength-dependence in the *K_1_* and K_2_ terms has been reported previously in studies at visible wavelengths (e.g., Virkkula et al., 2005; Virkkula, 2010; Ogren et al., 2017), but that dependence was relatively weak and introduced only small errors at the wavelengths studied. It is not clear whether the wavelength-dependence of the corrections can be neglected in the UV. However, we anticipate that our measured *σ_atn_* spectra are self-consistent and hence, fitting the measured channels that match the TAP channels to scale the entire spectrum is unlikely to introduce large errors.

*-dependence*. Another concern is the role of *ω* in corrections for multiple scattering in filter measurements. Unlike the particle suspensions in water used to obtain reference spectra for ocean color studies, reference measurements for atmospheric aerosol absorption rely on either photoacoustic methods that measure absorption directly or on subtracting measurements of scattering from extinction. The latter is particularly sensitive to errors when *ω* values are large, as absorption will be the small difference between large numbers. Virkkula et al. (2005) and Virkkula (2010) include *ω* in their correction scheme. Müller et al. (2014) used a constrained two-stream radiative transfer model approach to address the problem of reducing the errors in absorption filter measurements when *ω* is large. Ogren et al. (2017) used field measurements from 8 sites to show that they observed the lowest uncertainties (<35%) in the TAP measurements with greater absorption and lower *ω*. Conversely, they found much higher uncertainties when absorption was low and *ω* was high (up to ~100%). The uncertainty arising from *ω* is coupled to the uncertainties in the *K* values, with the results shown in Ogren et al. (2017) largely arising from uncertainties in the *K* parameters.

In the Ogren et al. (2017) study they combined all three channels in their evaluation of the uncertainties and observed median *ω* at their 4 low *ω* sites ranging from ~0.90 to ~0.92 versus the high *ω* subset with medians ranging from ~0.92 to ~0.97. The highly absorbing nature of the smoke aerosols observed here suggest that scattering errors are unlikely to be large. For all but the Nethker fire *ω* ≤ 0.92 was observed across the full wavelength range measured. Nethker, a predominantly smoldering fire (see Section 3.1 of the main text) exhibited *ω* ~0.95-0.98 over the 0.55 – 0.70 µm range, while at 0.35 µm *ω* ranged from ~0.73 to ~0.88. The positive curvature of the total absorption spectra combined with the negative curvature of extinction (see Fig. 4 and Section 3.2 of the main text) suggests that the scattering errors are likely to diminish in the UV range for the spectral data reported here.

Calibrating and developing suitable correction schemes for these hyperspectral total absorption spectra is a nontrivial problem. To our knowledge, there is no currently available hyperspectral scattering instrument that we can pair with our hyperspectral extinction instrument for the extinction minus scattering approach. Nor are there hyperspectral photoacoustic instruments available for this kind of experiment. Nonetheless we hope the data shown here will motivate new approaches that will improve our ability to quantitatively measure hyperspectral absorption. These data should be viewed with these caveats and this goal in mind.

**Section S6.** Histograms of linear and second-order polynomial fit residuals for entire data set

The residuals from both the linear and second-order polynomial fits were binned into 20 bins. The wider bins of the linear fit histograms (black bars, Fig. S10) indicate a wider range in values for the residuals (= measured spectrum – fit spectrum). The greater number of counts in the bins near zero for the second-order polynomial fits (red bars, Fig. S10) indicate a better fit to the measured spectrum. See the discussion in the main text regarding the more limited improvement for *σ_DI-abs_* compared to the rest of the measured spectra set.

Figure S10. Comparison of residuals from linear fits (black) and second-order polynomial fits (red) for all spectra in each set: a) *σ_ext_*, b) *σ_abs_*, c) *σ_MeOH-abs_*, d) *σ_DI-abs_*. Note, the fits were applied to the LN(0.3 – 0.7 µm) wavelength range for all but *σ_abs_* which was limited to LN(0.35-0.7 µm).

**Section S7.** Spectral difference between aerosol- and solution-phase of nigrosine

Differences in the spectrum of nigrosine (Fig. S11) were found when comparing aerosol-phase measurements in tests of the *σ_abs_* system to those obtained during the KORUS-OC study when testing the *σ_DI-abs_* LWCC measurement system. The aerosols were nebulized from a concentrated DI solution (visibly dark purple) and collected on a filter for measurement as described in Section S5 above. The bulk solution for the LWCC measurement was dilute with little discernible color. As noted in the main text, no ancillary measurements were made of pH so we cannot confirm the source of the difference. Some of the spectral differences are subtle, such as the shift to a longer wavelength from where the minimum occurs in the solution absorbance to that in the aerosol attenuation (from ~0.42 µm to ~0.43 µm) or the slightly narrower mid-visible primary peak in the former (maximum at ~0.56 µm) than the latter (maximum ~0.57 µm). But there is a clear difference in the wavelength range and amplitude of the secondary peak in the solution absorbance (~0.62-0.65 µm, maximum at 0.63 µm) than the aerosol attenuation (~0.64-0.68 µm, maximum at 0.66 µm).

Figure S11. Comparison of measured absorbance (solution phase) via LWCC (blue curve, right axis) and attenuation (aerosol phase) via filter-based measurement (yellow curve, left axis) of nigrosine in laboratory experiments.

**Section S8.** Partial spectra, curvature and axis of symmetry mapping in (*a_1_*,*a_2_*) coordinates

*Partial spectra.* As discussed in Jordan et al. (2021a, 2001b), spectral curvature leads to the result that the values of *α* or *a_1_* and *a_2_* will depend on the wavelength range of the measurement. This is well known to cause variations in *α* calculated using different wavelength pairs in the case of in situ measurements at individual wavelengths. A given wavelength range can be described by its “characteristic wavelength” (*****_ch_***), calculated from the relationship between the linear fit and the second-order polynomial fit to the logarithmically transformed spectrum via

$\lambda_{ch}=e^{{-\left( \alpha+a_{1} \right)}/\left( 2a_{2} \right)}$ (Eq. S1)

For a measurement over a given wavelength range, there is little variability in *λ_ch_*. However, for cases where some part of the measured spectral range is below detection at longer wavelengths (as seen here in *σ_DI-abs_* in Fig. 4b of the main text), the partial spectrum represents a shorter *λ_ch_* leading to a shift in its (*a_1_*,*a_2_*) mapping (see Figs. 9b and S8 in Jordan et al. (2021a)). It can also result in extreme values for *a_1_* and *a_2_* as shown here in Fig. S12a and b, e.g., |*a_1_*| > 10.

Figure S12. Comparison of (*a_1_*,*a_2_*) values and maps from complete *σ_DI-abs_* spectra measured over 0.35 – 0.7 µm (bottom panels) to those including partial spectra (top panels). The (*a_1_*,*a_2_*) data points shown in each panel are colored either by the axis of symmetry of the second-order polynomial fit (left panels) or by the* α_DI-abs_* (right panels).

In the main text, (*a_1_*,*a_2_*) maps are shown separately for each spectra set in order to highlight differences for a given type of measurement. In Fig. S13 all measurements are plotted together for comparison. For this exercise, only the complete *σ_DI-abs_* spectra over LN(0.35-0.7 µm) are included. First, this figure will be used to illustrate how to interpret (*a_1_*,*a_2_*) mapping space. Second, a couple of key takeaways from the comparison plot will be noted.

*Curvature and Axis of Symmetry*. For a given *λ_ch_*, observed ambient spectra fall into a relatively narrow range of values in (*a_1_*,*a_2_*) space (see Figs. 9b and S8 in Jordan et al. (2021a)) generally ranging from the positive *a_1_* and *a_2_* quadrant to the negative *a_1_* and *a_2_* quadrant (Fig. S13). Interpreting the mapping is straightforward for the y-axis (*a_2_*): larger absolute values of *a_2_* indicate greater curvature, whether positive (absorption spectra) or negative (extinction spectra). However, the distribution along the x-axis (*a_1_*) is less intuitive. Recall that a second-order polynomial describes a parabola (here, in terms of LN(*λ*) and *σ_p_*, where *p* represents one of the optical properties). The parameters that describe a parabola are curvature (*a_2_*) and its axis of symmetry (where LN(*λ*) = -*a_1_*/2*a_2_*). Hence, the x-axis can be understood as capturing shifts in the axis of symmetry to longer or shorter wavelengths for a given curvature *a_2_*. However, this calculation requires caution as *a_2_* approaches zero, e.g., the large negative value (~ -150 = LN(*λ*), or *λ*=7E-66 µm) for the axis of symmetry determined for one of the partial spectra (blue point in Fig. S12a) arises due to essentially dividing by zero.

For the positive curvature cases, the minimum axis of symmetry for the total absorption spectra was -0.38 (or *λ* = 0.68 µm) and for the soluble absorption spectra (both MeOH and DI) it was -0.23 (or *λ* = 0.79 µm). All other values are larger, therefore shifting to longer wavelengths. Maximum values were 0.002 (*σ_abs_*; *λ*=1.002 µm), 0.580 (*σ_MeOH-abs_*; *λ*=1.786 µm), and 2.382 (*σ_DI-abs_*; 10.82 µm). Hence, as shown in Fig. S12c and Fig. S13a, the axis of symmetry shifts to longer wavelengths from positive values of *a_1_* to more negative values of *a_1_*, and from larger values of *a_2_* to smaller values of *a_2_*. Note, do not take the values of the axis of symmetry literally. These spectra are not in fact parabolas. This discussion is intended to explain how to interpret the mapping in (*a_1_*,*a_2_*) space. Second-order polynomials better capture the observed spectral curvature than linear fits over the observed wavelength range, but both are empirical

relationships not derived from first principles of underlying physics or chemistry, and extrapolating the fit too far beyond the measurement range can lead to large errors.

In contrast, for the negative curvature spectra (extinction), the spectra exhibit less curvature than the absorption spectra and hence, are found closer to the *a_2_* = 0 line (Fig. S13). Where the axis of symmetry for the positive curvature absorption spectra was typically found at wavelengths longer than the maximum wavelength measured, the opposite is found here, with the axis of symmetry located at wavelengths shorter than the minimum wavelength measured. For *σ_ext_*, the minimum value for the axis of symmetry (-6.43, or *λ*=0.002 µm) is found for the spectrum exhibiting the least curvature (dark blue point, Fig. S13a) and the maximum value (-1.63, or *λ*=0.196 µm) is found for the spectrum exhibiting the greatest curvature. In this quadrant, the axis of symmetry shifts to longer wavelengths as both *a_1_* and *a_2_* shift to more negative values (Fig. S13a).

*Key Observations*. First, the curvature of *σ_ext_* is negative for all the measurements here. This is to be expected as the filters were all sampling behind a PM_2.5_ cyclone, i.e., there was no coarse fraction sampled that might have resulted in positive curvature. Second, given the observation in Figs. 4, 6, and S11 that bulk solution absorption spectra may shift spectral features from what one would observe from the aerosol phase, the differing curvature across the absorption spectra sets shown in Fig. S13 suggests it would be valuable to begin to routinely measure aerosol-phase absorption spectra rather than reconstructing such spectra from bulk solution absorption. At present the only method available to measure hyperspectral aerosol-phase absorption from 0.3-0.7 µm that we are aware of relies on the filter methodology shown here. Development of other techniques that eliminate the need for its challenging corrections (see Section S5) would be helpful.

Figure S13. Comparison across the LN(0.35-0.70 µm) fits to the 4 measured spectra sets: *σ_ext_*, filled circles, *σ_abs_*, filled triangles, *σ_MeOH-abs_*, asterisks, and *σ_DI-abs_*, filled diamonds. Data points colored by a) the axis of symmetry, and b) the Ångström exponent, *α*.

**Section S9.** Additional examples of spectral features evident in *σ_DI-abs_* spectra

Spectral features related to chromophores are evident in *σ_DI-abs_* spectra (Fig. S14) as well as in the *σ_MeOH-abs_* spectra shown in Fig. 9 of the main text. The relative importance of features at different wavelengths is observed to differ between fires (Fig. S14) and to some extent between samples of a given fire (not shown).

Figure S14. Like Fig. 9, linear fits (black curves, left panels) and second-order polynomial fits (black curves, right panels) to measured LN(*σ_DI-abs_*(0.3-0.7 um)) spectra (red curves, all panels) are shown with residuals at the same scale for both fits (blue curves). Here, rescaling of the residuals is not needed to highlight spectral features. The 3 examples shown are the same as those in Figs. 4 and 5, except for the Little Bear example already provided in Fig. 6 (recall, there are no *σ_DI-abs_* spectra from Williams Flats). The x-axis labels in wavelength units of µm shown along top panels for convenience.
